# The pentose phosphate pathway contributes to excess lactate production in radiation-induced fibroblast to myofibroblast transdifferentiation

**DOI:** 10.1016/j.jbc.2026.113142

**Published:** 2026-05-13

**Authors:** Josly Pierre-Louis Odoom, Sarah V. Camus, Mark Sfeir, Yang Yue, L. Ashley Cowart, Margaret A.T. Freeberg, Thomas H. Thatcher, Patricia J. Sime

**Affiliations:** 1 Department of Internal Medicine, Virginia Commonwealth University, Richmond, Virginia, USA; 2 Department of Biochemistry and Molecular Biology, Virginia Commonwealth University, Richmond, Virginia, USA; 3 Richmond Veterans Affairs Medical Center, Richmond, Virginia, USA

**Keywords:** radiation-induced pulmonary fibrosis, metabolic reprogramming, glycolysis, metabolic rewiring, pentose phosphate pathway, pulmonary fibrosis, primary human lung fibroblasts, metabolomics

## Abstract

Thoracic radiation is an effective mainstay treatment for lung cancer; however, patients risk developing an adverse side effect known as radiation-induced lung injury (RILI). RILI is dose-limiting, can be permanent, and may threaten normal lung function, but the underlying mechanism is not well characterized. RILI can include both inflammatory (radiation pneumonitis) and fibrotic (radiation-induced lung fibrosis) pathologies. Myofibroblasts are the main effector cells of fibrosis, and we and others have shown that ionizing radiation induces differentiation of normal lung fibroblasts to the myofibroblast phenotype (FMT, fibroblast to myofibroblast transdifferentiation). We previously reported that radiation induces production of excess lactate, which promotes an acidic microenvironment that activates the major profibrotic cytokine, transforming growth factor–β (TGFβ). Transforming growth factor–β in turn upregulates production of lactate, creating a profibrotic feed-forward loop. Here, we performed targeted metabolomics and metabolic tracer studies to determine how radiation alters cellular metabolism to promote fibrosis in cultured human lung fibroblasts and a mouse model of radiation induced lung fibrosis. Radiation upregulated both glycolysis and the pentose phosphate pathway (PPP), and we found that the PPP was a significant source of lactate production. Inhibition of glycolysis by targeting pyruvate kinase M2 prevented radiation-induced FMT and lactate production but did not affect fibronectin expression. However, when the gluconic shunt or the nonoxidative PPP is blocked by targeting glucose-6-phosphate dehydrogenase, FMT, lactate production, and fibronectin are markedly reduced. Our data reveal that the PPP is an important compensatory mechanism and driver of lactate accumulation observed in RILI.

Therapeutic thoracic radiation (TR) is a mainstay treatment for patients diagnosed with breast, esophageal, and lung cancer (1). Although effective at reducing cancer burden, ionizing radiation also damages healthy tissue, which can lead to short-term and long-term complications known as radiation-induced lung injury (RILI) (2). RILI can include both inflammatory (radiation pneumonitis) and fibrotic (radiation-induced pulmonary fibrosis) pathologies (2). Up to 80% of patients develop computed tomography (CT) detectable fibrotic changes to their lungs, while 5 to 20% experience clinical symptoms including shortness of breath, cough, and dyspnea (2). Mitigating the risk of RILI limits TR dosing, which may reduce the effectiveness of TR on decreasing tumor burden, especially in cases of cancer recurrence (3). RILI lesions on CT can make it more difficult to detect cancer recurrence, and patients with RILI are also at increased risk of respiratory infection (4, 5). Currently, there are no Food and Drug Administration (FDA)-approved treatments for RILI, although a phase 2 clinical trial of pirfenidone, currently only approved for idiopathic pulmonary fibrosis, showed promising results (6). An improved understanding of the underlying mechanisms involved in this potentially devastating side effect is critical before novel therapeutic targets can be identified.

We first identified an association between cellular metabolism and interstitial lung disease in idiopathic pulmonary fibrosis. We found that lung tissue, bronchoalveolar lavage (BAL) fluid, and exhaled breath condensate (EBC) from patients with idiopathic pulmonary fibrosis, and radiation-treated fibroblasts had elevated levels of lactate and the lactate-producing enzyme lactate dehydrogenase A (LDHA) (7, 8, 9). Excess lactate has also been reported in patients with other types of interstitial lung disease including sarcoidosis, asbestosis, and hypersensitivity pneumonitis (10, 11, 12). Elevated levels of secreted lactate are important because an acidic microenvironment can activate the latent form of the profibrotic cytokine transforming growth factor-β (TGFβ) (8). Transforming growth factor-β in turn promotes further fibroblast to myofibroblast transdifferentiation (FMT) with increased lactate production, resulting in a profibrotic feed-forward loop (13).

Pyruvate is the final product of glycolysis. From there, it can be converted to lactate, to acetyl-CoA for the tricarboxylic acid (TCA) cycle, or used in a variety of biosynthetic pathways including amino acid synthesis and gluconeogenesis (14). It is well-known that cancer cells modify their metabolism to promote aerobic glycolysis and lactate production, an observation known as the Warburg effect, and there is increasing evidence that something similar happens in pulmonary fibrosis (15), including a strong relationship between glycolysis, lactate, and pulmonary fibrosis (16, 17, 18). However, it is unlikely that the only metabolic changes associated with fibrotic lung injury are disruptions in lactate and glycolysis.

To investigate the broader spectrum of metabolic changes in radiation-induced pulmonary fibrosis, we performed metabolomic analysis on EBC from lung cancer patients with CT-confirmed fibrotic changes after TR. Compared to healthy controls or preradiation lung cancer patients, postradiation RILI cases exhibited a distinct metabolic signature, highlighted by increased lactate and concurrent changes in lipid, amino acid, and carbohydrate energy metabolism associated with the TCA cycle (19). Notably, our data supported TCA cycle deficiency and compensatory modes from alternative energy sources to meet the metabolic demands of chronic wound repair.

The pentose phosphate pathway (PPP) is parallel to glycolysis. Typically, the PPP takes glucose as an input to synthesize five carbon sugars, nucleotides, and NADPH *via* the gluconic shunt (20). Operating in reverse, the PPP can use five carbon sugars as input to provide intermediates to glycolysis (21). In irradiated cancer cells, both the forward and reverse PPP may be important. The forward PPP generates NADPH which is an important antioxidant and also required for regeneration of glutathione, both of which can lead to radioresistance in the irradiated tumors, whereas reverse flux through the PPP can generate additional energy from glycolysis (22, 23, 24).

Here, we performed metabolomic analysis on irradiated primary human lung fibroblasts (HLFs) and lung tissue from a mouse model of radiation fibrosis to investigate the interaction between glycolysis and the PPP in RILI. We found that irradiation increased both glycolysis and PPP, and that ribose was a significant precursor of upregulated lactate production. Inhibition of glycolysis blocked FMT but not expression of matrix protein fibronectin in HLFs, while inhibition of reverse PPP flux blocked both FMT and fibronectin expression. Thus, the PPP is an important compensatory mechanism and driver of lactate accumulation after thoracic irradiation.

## Results

### Targeted metabolomics of irradiated lungs identifies changes in energy metabolic pathways

**Figure 1:**
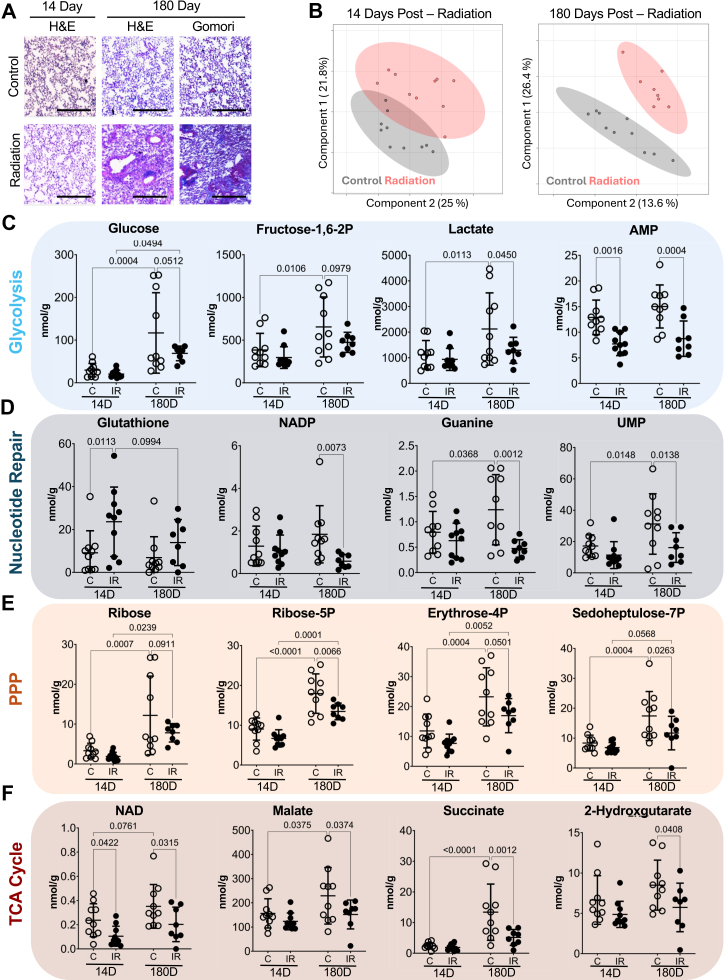
**Key energetic and synthetic metabolic pathways induced in C57BL/6J mice 14 days and 180 days after thoracic radiation treatment.** Groups of mice were irradiated as described, and lungs were harvested at 14 or 180 days after irradiation. *A*, representative H&E and Gomori trichrome. The scale bar represents 500 μm. *B*–*F*, targeted metabolomics was performed on tissue homogenates from the control or irradiated lungs. *B*, PLS-DA plot visualizing the overall distribution of mice based on targeted metabolomic profiles 14 and 180 days after irradiation. *C*–*F*, selected metabolites are shown, grouped according to their KEGG pathway assignment. *C*, glycolysis pathway metabolites. *D*, nucleotide repair pathway metabolites. *E*, pentose phosphate pathway (PPP) metabolites. *F*, TCA cycle metabolites. C = control lungs and IR = irradiated lungs. Values represent mean ± SD. *p* Values from two-way ANOVA. N = 8 to 10 mice per group. KEGG, Kyoto Encyclopedia of Genes and Genomes; PLS-DA, partial least-squares discriminant analysis; TCA, tricarboxylic acid.

To identify changes in metabolism in early stage and late-stage RILI, we analyzed mouse lung tissues from a radiation fibrosis model (25, 26). We performed targeted irradiation of the right lung lobe, at a dose of 6 Gy per day for five consecutive days. We chose a fractionated scheme to more closely model clinical irradiation strategies, with five fractions of 6 Gy calculated to have the same bioequivalent dose (BED) as a single session at 15 Gy, a model that has been previously reported (27). Mice were harvested 14 (inflammatory phase) and 180 days (fibrotic phase) after irradiation (Fig. S1). Histological staining of fixed sections revealed little change on day 14, while the day 180 sections show inflammatory infiltration with patchy fibrosis (Fig. 1*A*). The identified fibrosis is heterogeneous, with more dense areas where there are more collagen deposits. There were no significant changes in bronchoalveolar cell populations at day 14; however, lymphocytes were significantly increased at day 180 (Fig. S2).

Based on our findings of disrupted metabolism in exhaled breath condensate from lung cancer patients who developed RILI post irradiation (19), we preselected several metabolic pathways for analysis using a targeted metabolomics approach, including glycolysis, nucleotide repair, the PPP, and the TCA cycle. The metabolites and pathways are listed in Table S1. Partial least-squares discriminant analysis shows that mice cluster based on treatment, with separation and minimal overlap at day 14, and this separation became more pronounced at day 180 (Fig. 1*B*). Within the glycolysis intermediates, we found that older mice (day 180 control) had significantly higher concentrations of glucose, fructose-1,6-2P, and lactate compared to day 14 controls, while the concentration of these metabolites significantly decreases after radiation on day 180 (Fig. 1*C*). Adenosine monophosphate (AMP) content, an energy sensor molecule, is also reduced by irradiation on both days 14 and 180 (Fig. 1*C*).

Radiation also alters metabolites involved in nucleotide repair of single and double strand breaks. On day 14, the concentration of glutathione, which provides reducing equivalents to neutralize free radicals, is significantly high in irradiated lung tissue (Fig. 1*D*). On day 180, there remains an upward trend. After radiation, nicotinamide adenine dinucleotide phosphate (NADP), guanine and uridine monophosphate are elevated in older mice but are depressed in irradiated mice (Fig. 1*D*). Following the same pattern as glycolysis-associated metabolites, PPP intermediates, including ribose-5P, erythrose-4P, and sedoheptulose-7P, increase with age (Fig. 1*E*) but are depressed in irradiated mice at day 180. Radiation also altered the concentration of TCA cycle intermediates during early stage and late-stage RILI (Fig. 1*F*). The concentration of malate, succinate, and 2-hydroxyglutarate was significantly low after radiation on day 180. Although histological changes were noted only during the late stage time point, metabolic changes were identified early during RILI development.

### Radiation induces FMT and accelerates energy metabolism

**Figure 2:**
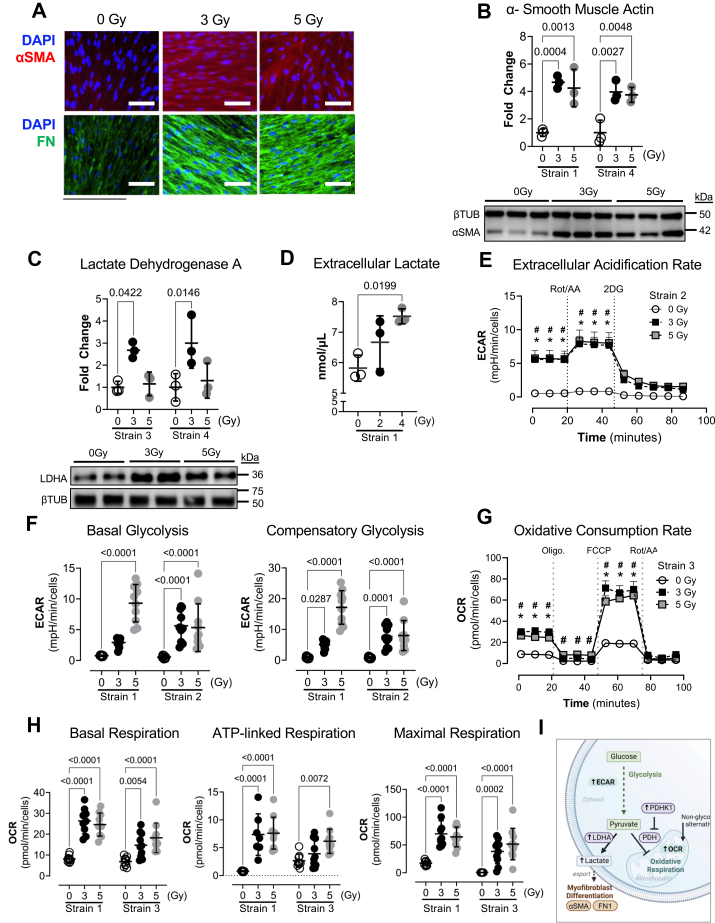
**Radiation induces myofibroblast differentiation, enhances lactate secretion, and accelerates glycolysis and cellular respiration in primary human lung fibroblasts (HLFs).** Primary HLFs were subjected to 0, 3, or 5 Gy irradiation, and harvested 5 days later. *A*, HLFs were immunostained for α-SMA (*red*) or fibronectin (FN, *green*), and counter-stained with DAPI (*blue*). The scale bar represents 100 μm. Western blot of (*B*) α-SMA or (*C*) LDHA protein expression, normalized to β-tubulin. Mean ± SD for N = 3 replicates is shown. *p* Values from one-way ANOVA with Dunnett’s MC test. *D*, lactate was determined in the supernatant from HLFs irradiated as indicated. Mean ± SD for N = 3 replicates is shown. *p* Values from one-way ANOVA, Dunnett’s MC test. *E*, extracellular acidification rate (ECAR) measured by Seahorse Xfe glycolytic rate assay in HLFs treated with 0, 3, or 5 Gy radiation, N = 8 replicates per condition. Values are mean ± SEM. One-way ANOVA, with Dunnett’s MC test, *p* ≤ 0.05. [0 versus 3 Gy(∗), versus 5 Gy(#)]. Rot/AA (rotenone/antimycin A) and 2DG (2-deoxyglucose). *F*, basal glycolysis and compensatory glycolysis are shown for two independent seahorse assays performed on HLFs from different donors. *G*, oxygen consumption rate (OCR) measured by Seahorse Xfe mitochondrial stress test assay in HLFs treated with 0, 3, or 5 Gy radiation, (n = 8). Values represent mean ± SEM. One-way ANOVA, Dunnett’s MC test *p* ≤ 0.05. [0 versus 3 Gy(∗), versus 5 Gy(#)]. FCCP (carbonyl cyanide-4-trifluoromethoxy phenylhydrazone). *H*, basal respiration, ATP-linked respiration and maximal respiration are shown for two independent Seahorse assays performed on HLFs from different donors. ATP-linked respiration is basal respiration minus suppressed respiration after adding oligomycin. *p* Values from one-way ANOVA with Dunnett’s post test. *I*, graphical summary showing the effects of radiation treatment on glycolysis, respiration, and lactate production in HLFs. Prepared with Biorender.com. LDHA, lactate dehydrogenase A; DAPI, 4′,6-diamidino-2-phenylindole; HLF, human lung fibroblast; αSMA, α-smooth muscle actin; MC, multiple comparison.

Myofibroblasts are the effector cells in fibrosis responsible for much of the excess extracellular matrix deposition (28, 29). To determine whether cellular metabolic reprogramming plays a role in radiation-induced myofibroblast differentiation, we irradiated HLFs to various doses of radiation and measured profibrotic and metabolic markers, compared to nonirradiated controls. To promote patient centricity and biological diversity, we conducted our *in vitro* experiments using HLFs derived from six different donors (See Table S2). As previously reported (8), ionizing radiation induced FMT, shown by increased expression of the differentiation marker α-smooth muscle actin (αSMA) and the matrix protein fibronectin by both immunocytochemistry (Fig. 2*A*) and western blot (Fig. 2*B*). Also consistent with previous reports (30), radiation increased the expression of the lactate-producing enzyme LDHA (Fig. 2*C*) and production of extracellular lactate in the medium (Fig. 2*D*).

Glycolysis and the TCA cycle are two major energy pathways that were enriched in the metabolome of the RILI mouse model. Thus, we hypothesized that glycolysis is an important mechanism in radiation-induced myofibroblast differentiation. To test the effects of radiation on glycolysis, we performed a series of bioanalyses using the Seahorse XFe system. The Seahorse XF Glycolysis Rate Assay measures the extracellular acidification rate as a proxy of glycolysis flux with or without mitochondrial-assisted acidification. The assay measures basal glycolysis, followed by the addition of rotenone and antimycin A (Rot/AA) which inhibits mitochondrial function allowing measurement of compensatory glycolysis. Finally, 2-deoxy-D-glucose (2-DG) inhibits all glycolysis and is a verification check for the assay (Fig. 2*E*) (31). Irradiated fibroblasts exhibit both increased basal and compensatory glycolysis compared to nonirradiated controls (Fig. 2, *E* and *F*).

To determine the effects of radiation on oxidative phosphorylation (respiration), we used the Seahorse XF Mito Stress Test Assay. Similar to the glycolysis assay, the Mito Stress Test uses the oxygen consumption rate (OCR) as a proxy for oxidative phosphorylation. The assay measures basal OCR, followed by addition of oligomycin, an ATP synthase inhibitor, which allows measurement of non-ATP-linked respiration. Addition of carbonyl cyanide-p-trifluoromethoxy phenylhydrazone (FCCP) uncouples respiration from ATP synthesis, allowing determination of maximal respiration. Finally, the addition of Rot/AA stops all respiration and is a control to validate the assay (Fig. 2*G*). Irradiated HLFs increased basal respiration, increased ATP-linked respiration and increased maximal respiration (Fig. 2*H*), suggesting increased numbers of mitochondria in irradiated HLFs, increased activity of the electron transport chain, or both. However, since our data also suggest preferential hydrogenation of pyruvate to produce lactate rather than acetyl-CoA (Fig. 2*I*), this raises the question of how HLFs support increased oxidative respiration.

### Radiation induces glycolytic reprogramming decreasing glucose utilization for energy

**Figure 3:**
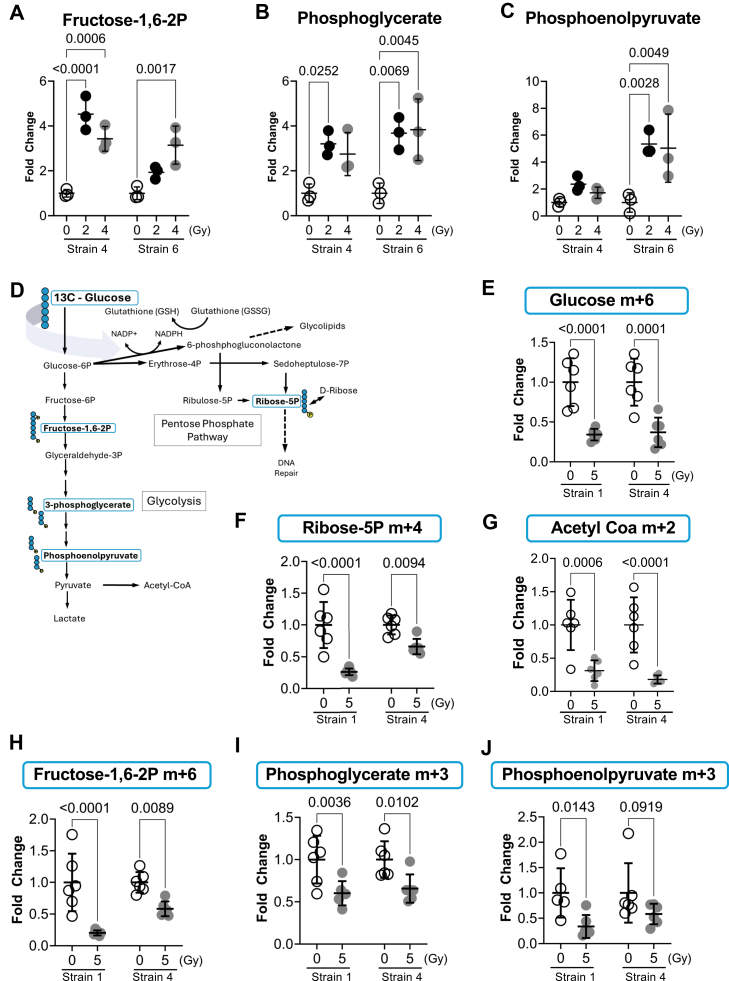
**U-^13^C-Glucose stable isotope tracing and targeted metabolomics reveal that radiation reprograms glycolysis.***A*–*C*, HLFs were irradiated as indicated, and cell lysates were submitted for targeted metabolomics. Selected rate-limiting glycolytic intermediates are shown. Values are mean ± SD for N = 3 replicates per cell strain, two donors (strains) per experiment. *p* Values are from one-way ANOVA with Dunnett’s MC test. *D*, schematic for the first-pass metabolism of uniformly labeled ^13^C-glucose carbons to the glycolysis and pentose phosphate pathways. *Solid circles* indicate heavy carbons. *E*–*J*, HLFs were irradiated as shown and harvested after 5 days. The standard medium was replaced with medium containing U-^13^C-glucose as the only sugar source, 4 h before harvest. The indicated metabolites were determined in cell lysates by LC-MS/MS. m + 2, m + 3, m + 4 and m + 6 indicate metabolites with increased mass due to the incorporation of 1 to 5 heavy carbons, as shown in A. Data shown are mean ± SD for six replicates per condition and strain. *p* Values from *t* test. LC-MS/MS, liquid chromatography-tandem mass spectrometry; HLF, human lung fibroblast; MC, multiple comparison

To better understand the role of accelerated glycolysis in the radiofibrogenic phenotype, we performed targeted metabolomics on lysates from irradiated and control HLFs. The concentration of glycolytic intermediates was significantly high after radiation treatment, including key rate-limiting metabolites fructose-1,6-2P, phosphoglycerate, and phosphoenolpyruvate (PEP) (Fig. 3, *A*–*C*).

Prior reports in patients with pulmonary fibrosis have reported an increased uptake of glucose associated with disease severity as detected by 18-fluorodeoxyglucose positron emission tomography (PET) scan (32, 33). To investigate whether increased glucose consumption by the glycolysis pathway was the source glycolytic reprogramming in irradiated HLFs, we performed a stable isotope tracing experiment using uniformly labeled [^13^C]-glucose as the only sugar source in the medium. The predicted first-pass metabolites with increased mass are shown in Figure 3*D*. M+4 to 6 refers to downstream metabolites that have glucose derived heavy carbons incorporated. Labeled glucose was significantly reduced in lysates from irradiated HLFs compared to controls (Fig. 3*E*). An alternative route for glucose carbons to metabolize is through the oxidative PPP to produce ribose-5P for nucleotide synthesis and other biosynthetic pathways, however, the concentration of label ribose-5P was also significantly reduced after irradiation (Fig. 3*F*). The contribution of glucose to the TCA cycle was determined by measuring labeled acetyl-CoA, which is significantly decreased in irradiated HLFs (Fig. 3*G*). The concentrations of labeled glycolysis intermediates fructose-1,6-2P, 3-phosphoglycerate (3PG), and PEP were also significantly lower in irradiated HLFs (Fig. 3, *H* and *I*). (Note that pyruvate and lactate were included in the targeted metabolomic panel but were below the limit of quantitation in this experiment. Thus, although radiation significantly accelerates glycolysis flux and accumulation of glycolytic intermediates, glucose does not appear to be the source for these intermediates.

### Radiation-induced myofibroblasts leverage the pentose phosphate pathway

**Figure 4:**
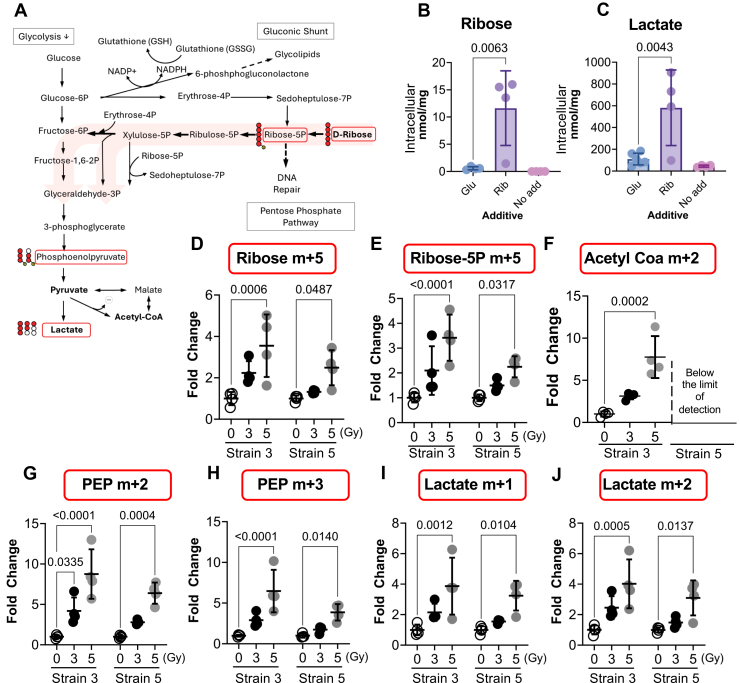
**U-^13^C-Ribose stable isotope tracing and targeted metabolomics traces radiation-induced lactate production and glycolysis back to ribose.***A*, schematic for the first-pass metabolism of uniformly labeled ^13^C-ribose carbons to the glycolysis and pentose phosphate pathways. *Solid circles* indicate heavy carbons. *B*–*C*, nonirradiated HLFs were treated with unlabeled glucose or ribose for 4 h prior to harvest and analysis. Intracellular ribose and lactate concentrations are shown. Values shown are mean ± SD for N = 4 replicates per treatment. *p* Values from one-way ANOVA with Dunnett’s MC test. *D*–*J*, HLFs from two different donors were irradiated as indicated and harvested after 5 days. Four hours before harvest, the standard medium was replaced with medium containing uniformly labeled ^13^C-ribose as the only sugar. Lysates were analyzed by targeted metabolomics. Selected metabolites are shown. m + 1, m + 2, m + 3, and m + 5 indicate metabolites with increased mass due to the incorporation of 1 to 5 heavy carbons, as shown in A. Values shown are mean ± SD for N = 4 replicates per treatment. *p* Values from one-way ANOVA with Dunnett’s MC test. HLF, human lung fibroblast; MC, multiple comparison.

The PPP runs in parallel with glycolysis and typically converts 6-carbon sugars into 5-carbon sugars for nucleotide synthesis and other biosynthetic pathways (Fig. 4*A*). However, the PPP can also operate in reverse, using 5-carbon sugars for energy *via* glycolysis and the TCA cycle, and this reverse PPP mechanism has been demonstrated in cancer cells (22). Therefore, we hypothesized that the PPP may be an alternative carbon source to the glycolysis pathway to compensate for the loss of glucose to lactate production and export. First, to test whether HLFs could take up exogenous ribose, nonirradiated HLFs were incubated with glucose-free Dulbecco's modified Eagle's medium (DMEM) (no added sugar), glucose free-DMEM containing 5 mM ribose, or DMEM containing 5 mM glucose for 4 h prior to lysis and targeted metabolomic analysis. There was a significant amount of intracellular ribose in the ribose-additive group indicating ribose uptake by HLFs (Fig. 4*B*). To our surprise, trading glucose for ribose in the medium also resulted in a significant increase in intracellular lactate (Fig. 4*C*). To understand the effects of radiation on ribose utilization by glycolysis, irradiated and control HLFs were incubated with uniformly labeled [^13^C]-ribose for 4 h prior to harvest, then lysates were subjected to targeted metabolomics analysis. The concentrations of labeled ribose and labeled ribose-5P are elevated after irradiation, confirming uptake of the labeled ribose and that it was incorporated into the PPP (Fig. 4, *D* and *E*). Irradiated HLFs also exhibited increased amounts of labeled PEP and acetyl-CoA, indicating entry of labeled ribose into the glycolysis pathway and its use for oxidative phosphorylation in the TCA cycle (Fig. 4, *F*, *G* and *J*). The concentration of labeled lactate was also significantly higher in irradiated HLFs (Fig. 4, *H* and *I*). These data suggest that irradiated HLFs undergo metabolic reprogramming to use the PPP as a compensatory energy source that drives lactate production and respiration.

### Irradiation increases the ribose salvage pathway

**Figure 5:**
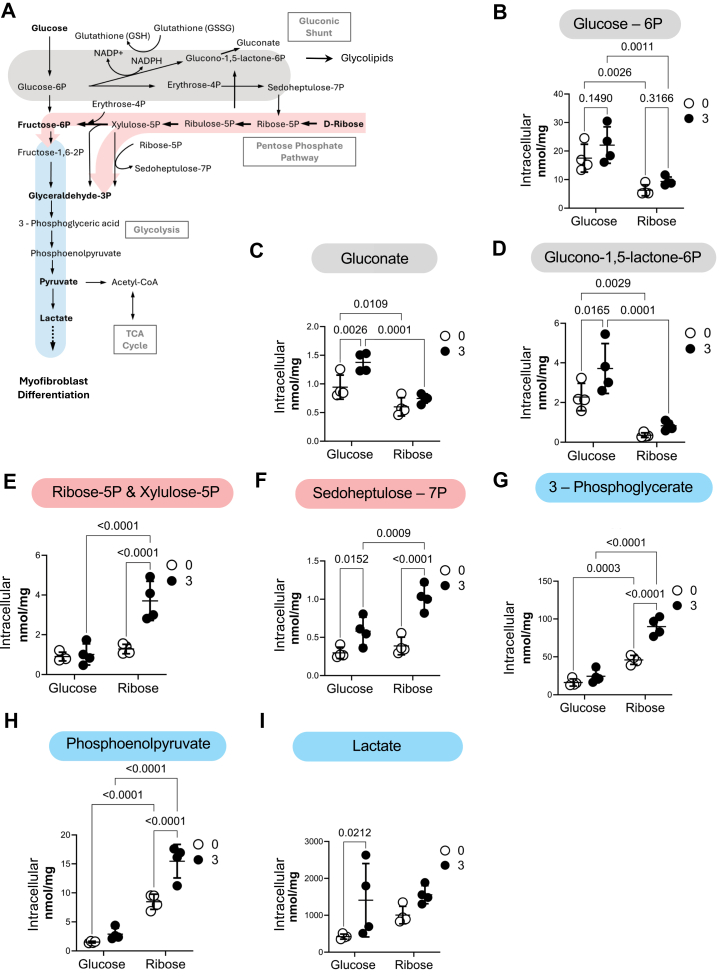
**Radiation drives anabolic utility of glucose through the gluconic shunt (oxidative PPP) in HLFs.***A*, schematic for the flow of glycolysis and compensatory alternate pathways, the gluconic shunt, and the PPP. The gluconic shunt is highlighted in *gray*, the PPP in *pink*, and glycolysis in *blue*. *B*–*J*, HLFs were irradiated as indicated and harvested on day 5. Four hours prior to harvest, the standard medium was removed and replaced with DMEM containing either 5 mM glucose or 5 mM ribose. After harvest, lysates were submitted for targeted metabolomics analysis. *B*, the intracellular concentration of gluconic shunt intermediates glucose-6-phosphate, (*C*) gluconate, and (*D*) glucono-1,5-lactone-6-phosphate; PPP intermediates (*E*) ribose-5-phosphate and xylulose-5-phosphate compound (*F*) sedoheptulose-7-phosphate; glycolysis intermediates (*G*) 3-phosphoglycerate, (*H*) phosphoenolpyruvate, and (*I*) lactate. Data means are SD for N = 4 replicates. *p* Values from two-way ANOVA with uncorrected Fisher’s LSD post test. DMEM, Dulbecco's modified Eagle's medium; HLF, human lung fibroblast; LSD, least significant difference; PPP, pentose phosphate pathway.

To better understand the metabolic alterations in glycolysis and the PPP after irradiation, irradiated HLFs were maintained in glucose-containing medium, or switched to ribose-containing medium for 4 h before harvest, with lysates submitted for targeted metabolomics analysis. Because the PPP is biphasic, we included metabolites involved in oxidative PPP (the gluconic shunt) and nonoxidative PPP phase (Fig. 5*A*). Irradiation does not alter the concentration of glucose-6P, but glucose-6P levels are significantly low with ribose as the sugar source (Fig. 5*B*). Gluconate and glucono-1,5-lactone-6P are significantly elevated in HLFs using glucose compared to ribose, and accumulate even more with irradiation (Fig. 5, *C* and *D*). This suggests that when glucose is the available sugar, it is used for synthesis of glutathione and glycolipids *via* the gluconic shunt. Ribose-5P and xylulose-5P are increased in irradiated HLFs using ribose but not glucose (Fig. 5*E*). Sedoheptulose-7P accumulates after irradiation under both sugar conditions; however, the increase is higher with ribose (Fig. 5*F*). Glycolysis and the PPP can exchange sugars at two points, xylulose-5P↔fructose-6P and xylulose-5P↔glyceraldehyde-3P. Fructose-6P and glyceraldehyde-3P were not detected, likely because they are converted rapidly to fructose-1,6-2P and 3-phosphoglycerate, respectively (34, 35). 3-PG and PEP were significantly increased in irradiated HLFs using ribose as their sugar source (Fig. 3, *H* and *I*). There is a trend toward increased lactate in irradiated HLFs using either glucose or ribose that is not significant in this experiment, but is consistent with the results in Figures 2*D* and 4*B*. Taken together, these data suggest that radiofibrogenic metabolism in HLFs may be characterized as a coordinated system involving glucose consumption for anabolic synthesis through the gluconic shunt and a compensatory mechanism of ribose for catabolic energy production.

### Pyruvate kinase M2 and the PPP are required for irradiation-induced FMT

**Figure 6:**
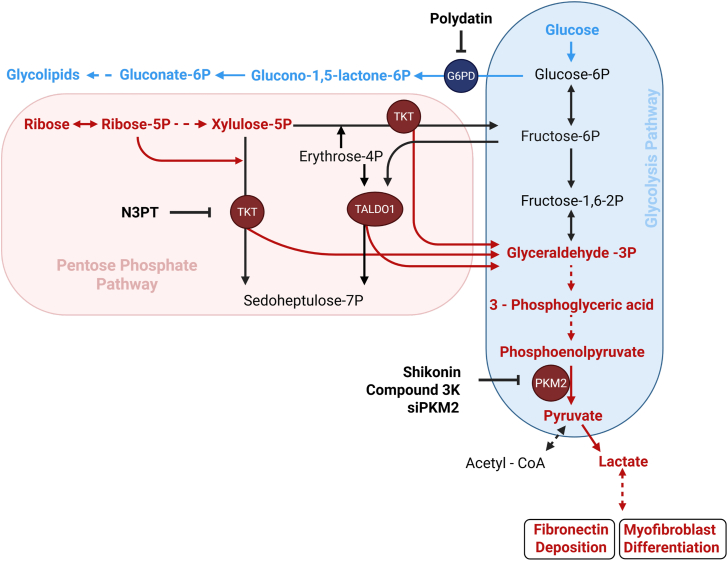
**Key steps in glycolysis and the pentose phosphate pathway.** Schema showing key steps in the glycolysis (*blue*), pentose phosphate pathway (*pink*), and the gluconic shut (*blue* text, showing the targets for inhibition). We hypothesize that increased conversion of pyruvate to lactate and the diversion of glucose to biosynthesis *via* the gluconic shunt is compensated for by the incorporation of five carbon sugars into glycolysis *via* the reverse PPP. G6PD, glucose-6-phosphate dehydrogenase; TKT, transketolase; PKM2, pyruvate kinase M2; N3PT, N3-pyridyl thiamine. Created in BioRender. Thatcher, T. (2026) https://BioRender.com/yo6vzz9. PPP, pentose phosphate pathway.

**Figure 7:**
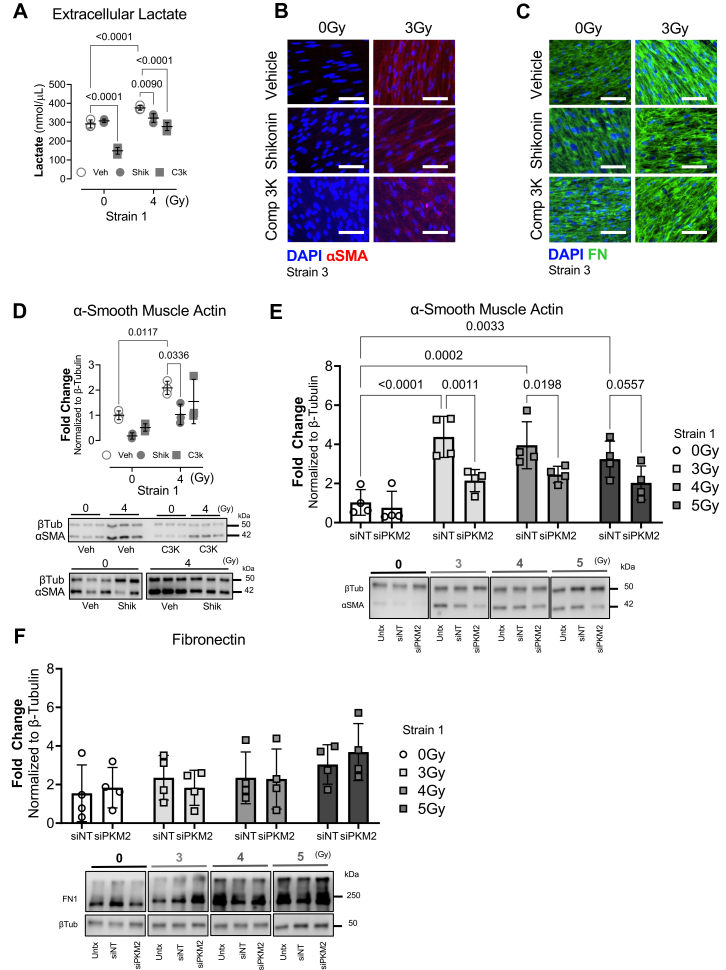
**Pyruvate kinase M2 is required for radiation-induced myofibroblast differentiation in HLFs.** HLFs were irradiated as indicated and cultured for 5 days before harvest. Cultures were pretreated with shikonin, compound 3K (C3K) or vehicle 1 h before irradiation and the compounds were maintained in the cell culture for the full 5 days. *A*, extracellular lactate was measured in conditioned medium. Data shown are mean ± SD for four replicate cultures. *p* Values from two-way ANOVA with Šídák’s post test. *B* and *C*, HLFs were immunostained for α-SMA (*red*) or fibronectin (FN, *green*) and counterstained with DAPI (*blue*). Representative images are shown. The scale bar represents 100 μm. The immunocytochemistry results were obtained in a single large experiment that tested multiple combinations of drugs and radiation doses. Images from cells treated without drugs are used in Figure 2, and images from the same wells are included as the no-drug controls in Figures 7 and 8. *D*, expression of α-SMA was determined by western blot, normalized to β-tubulin loading control. *E* and *F*, HLFs were transfected with an siRNA for PKM2, a control siRNA, or left untreated. Knockdown of PKM2 expression was confirmed by RT-PCR and by western blot (data not shown). Forty-eight hours after transfection, the HLFs were irradiated as indicated, and proteins were harvested on day 5 after irradiation. α-SMA and fibronectin expression were determined by western blot. The same blot was probed simultaneously for α-SMA, fibronectin, and β-tubulin loading control. Quantitation was performed as described and normalized to β-tubulin. *PanelE* shows the α-SMA and β-tubulin regions of the blot with quantitation, and *panelF* shows the fibronectin and β-tubulin region of the blot with quantitation. For *D*–*F*, data shown are mean ± S.D. for 3 to 4 replicates per condition. *p* Values from two-way ANOVA with Dunnett’s post test. αSMA, α-smooth muscle actin; DAPI, 4′,6-diamidino-2-phenylindole; HLF, human lung fibroblast; PKM2, pyruvate kinase M2.

Metabolomics analysis suggests that both glycolysis and the PPP are required for FMT, but as metabolomics only provides a systems-level “snapshot” of metabolism at one moment in time (36), we used pharmacologic and genetic approaches to confirm the importance of both glycolysis and the PPP. Pyruvate kinase M2 (PKM2) is the final enzyme in the glycolysis pathway, converting PEP to produce pyruvate (Fig. 6) (37). It is important to note that PKM2 can exist as a monomer, a homodimer, or a homotetramer, and only the tetramer is active in converting PEP to pyruvate (38). To better understand the role of PKM2 in the context of radiation-induced FMT, we targeted PKM2 with two different pharmacologic inhibitors, Compound 3K (C3K) and shikonin. C3K is a selective inhibitor for PKM2 (IC_50_ = 2.95 μM) that disrupts tetramerization and dimerization activity (39). Shikonin is a flavonoid derivative known to inhibit PKM2 activity by promoting macropolymer aggregation to an inactive form (39, 40). Addition of either C3K or shikonin to irradiated HLFs significantly reduced extracellular lactate accumulation compared to vehicle-treated HLFs (Fig. 7*A*). Immunocytochemistry analysis of irradiated HLFs show that both shikonin and C3K attenuated expression of the myofibroblast differentiation marker αSMA (Fig. 7*B*) but did not reduce radiation-induced expression of fibronectin (Fig. 7*C*). Western blot confirmed a significant reduction in radiation-induced FMT with shikonin and a downward trend with C3K (Fig. 7*D*). The importance of PKM2 was confirmed by siRNA knockdown. PKM2 knockdown by siRNA transfection reduced radiation induced FMT across three separate doses of radiation (Fig. 7*E*), but again, did not reduce expression of fibronectin (Fig. 7*F*).

**Figure 8:**
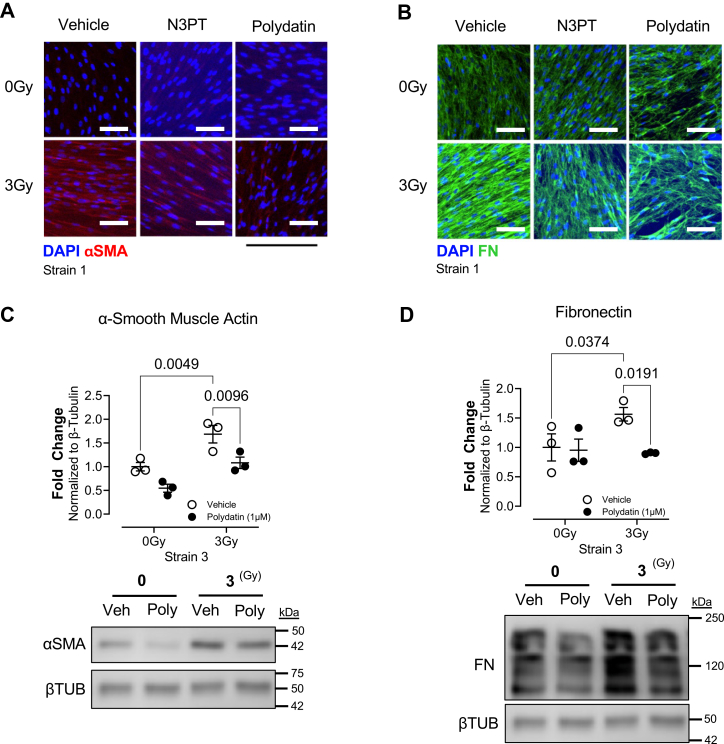
**The PPP by polydatin or N3-pyridyl thiamine suppresses radiation-induced FMT and fibronectin expression.** HLFs were irradiated as indicated and cultured for 5 days before harvest. Cultures were pretreated with polydatin or N3-pyridyl thiamine N3PT or vehicle 1 h before irradiation and the compounds were maintained in the cell culture for the full 5 days. *A* and *B* HLFs were immunostained for α-SMA (*red*) or fibronectin (FN, *green*) and counterstained with DAPI (*blue*). Representative images are shown. The scale bar represents 100 μm. The immunocytochemistry results were obtained in a single large experiment that tested multiple combinations of drugs and radiation doses. Images from cells treated without drugs are used in Figure 2, and images from the same wells are included as the no-drug controls in Figures 7 and 8. *C* and *D*, expression of α-SMA and fibronectin was determined by western blot. The same blot was probed simultaneously for α-SMA, fibronectin, and β-tubulin loading control. Quantitation was performed as described and normalized to β-tubulin. *PanelC* shows the α-SMA and β-tubulin regions of the blot with quantitation, and *panelD* shows the fibronectin and β-tubulin region of the blot with quantitation, normalized to β-tubulin loading control. Data shown are mean ± S.D. for three replicates per condition. *p* Values from two-way ANOVA with uncorrected Fisher’s LSD post test. PPP, pentose phosphate pathway; FMT, fibroblast to myofibroblast transdifferentiation; N3PT, N3-pyridyl thiamine; DAPI, 4′,6-diamidino-2-phenylindole; HLF, human lung fibroblast; αSMA, α-smooth muscle actin.

We also used pharmacologic inhibition to target the PPP. Polydatin, a glucose-6-phosphate dehydrogenase (G6PD) inhibitor (41), blocks the rate-limiting switch of glucose toward the gluconic shunt or the oxidative phase of the PPP, while N3-pyridyl thiamine (N3PT), a potent and selective transketolase (TKT) inhibitor (42), blocks ribose contribution to the glycolysis pathway through fructose-6P or glyceraldehyde-3P (Fig. 6). We confirmed with an activity assay that N3PT blocked TKT activity in HLF lysates (Fig. S3). HLFs were irradiated as described and treated with N3PT or polydatin. Immunocytochemistry staining and western blot analysis showed that both polydatin and N3PT treatment reduced radiation-induced expression of αSMA (Fig. 8, *A* and *C*). In contrast to inhibition of PKM2, inhibition of either G6PD or TKT with polydatin or N3PT also inhibited expression of the extracellular matrix protein fibronectin (Fig. 8, *B* and *D*). Thus, while glycolysis is necessary for FMT but not increased matrix expression in irradiated HLFs, the PPP is required for both FMT and increased matrix expression. Interestingly, neither polydatin nor N3PT inhibited the accumulation of extracellular lactate in the conditioned medium (Fig. S4). This could be due to several factors, including downregulation of lactate transporters preventing lactate from reaching the medium, consumption of lactate by the HLFs as an energy source, production of lactate by the normal glycolysis pathway.

## Discussion

Thoracic cancer patients who undergo radiation therapy risk developing RILI. When chronic, RILI results in localized scar tissue accumulation which can mask the presence of recurring tumors and heighten risk for infection (4). Pirfenidone, an antifibrotic approved for treating idiopathic pulmonary fibrosis, was investigated for treating RILI in lung cancer patients, resulting in improved lung function in patients with mild to moderate fibrosis and fewer acute exacerbations, although further studies are required for the use of pirfenidone for RILI (43). Notably, pirfenidone has severe side-effects, which result in 42% to 76% of patients being unable to take the full recommended dose, and 34% to 66% of patients discontinuing treatment entirely (44, 45, 46, 47). There is currently no Food and Drug Administration-approved cure for RILI, thus further investigation may provide mechanistic insights required to identify novel therapeutic targets.

To our knowledge, we were the first to identify that ionizing radiation increases lactate production in myofibroblasts, the key effector cells of fibrosis (8, 30). This discovery provided an initial clue for the significance of cellular metabolism in profibrogenesis. Targeting the lactate producing enzyme LDHA revealed its requirement for perpetuating a feed forward loop (48). We also investigated exhaled breath in RILI patients and identified striking largescale metabolic shifts that go beyond lactate (19). Here, we sought to determine how radiation alters cellular metabolism to promote fibrosis in cultured HLFs and a mouse model of radiation-induced lung fibrosis.

Consistent with EBC data in patients with RILI, our investigation in mice showed that radiation significantly alters cellular metabolism beyond lactate alone, including the TCA cycle, glycolysis, and the PPPs (Fig. 1). We previously reported that human exhaled breath from patients with RILI had elevated levels of lactate (19), and we show here that human lung fibroblasts/myofibroblasts produce more lactate after irradiation, but here, the mouse lung tissue contains lower levels of lactate. It is important to note that metabolomics only shows a snapshot of metabolites at a particular time point, and cannot, by itself, show the ebb and flow of metabolites across pathways. AMP was also decreased in the mouse lungs, which corresponds to elevated ATP and a highly energetic state overall (49), so the decrease of key glycolysis intermediates may reflect their rapid consumption. To the extent the changes in glycolysis in RILI are similar to the Warburg effect, we propose that lactate is being consumed for energy by regions of the fibrotic “tumor” that have less access to oxygen, with the difference between mouse and human due to the speed of disease progression, different metabolic needs of the tissues, or other intraspecies differences. Finally, the mouse lung metabolomics results were obtained from whole lung lysates. The emerging field of spatial metabolomics may shed more light on the metabolic state of different tissue types (epithelium, endothelium, fibroblastic foci, and so on) in the future. Indeed, spatially resolved metabolomics in the bleomycin and silica mouse models of lung fibrosis also identified disruption of glycolysis, TCA cycle, and purine/pyrimidine metabolism (50). Complex amino acids, TCA cycle, and nucleotide synthesis *via* the PPP, and pyruvate metabolism were enriched in a single-dose targeted TR model in Sprague-Dawley rats where lung tissue was collected 1 week after irradiation (51). Collectively, our data support that changes in cellular energetic and synthetic metabolism is a critical defining feature in the RILI response.

Progress has been made to understand how radiation acutely affects cellular metabolism *in vivo* (52, 53), but to our knowledge, we are the first to characterize radiation fibrometabolism at early and late phases in mice. Note there were metabolites below the limit of detection indicating that only trace amounts of that metabolite were present in the sample. Some biochemical reactions may occur very quickly causing those metabolites to get consumed. Still, we identified global changes in metabolism prior to measurable fibrotic changes in the tissue. Glycolysis and PPP-related metabolites were found to increase with age. Aging is a well-known component of fibrosis development (54, 55). Our data suggest that age may prime a metabolic niche susceptible to profibrotic rewiring. Our findings also support a time course untargeted metabolomic study conducted in serum of lung cancer patients who received radiation therapy (56). Metabolites involved in the TCA cycle, fatty acid beta-oxidation, and the cycling of nucleotides, were enriched in the serum of lung cancer patients who received radiation therapy and these metabolites were associated with acute/late radiation therapy toxicity (56). Thus, our mouse study provides further support and insight on which pathways beyond lactate are significant in the RILI response and potential therapeutic targets for the clinical setting. Collectively, cellular metabolism may offer prognostic utility for an RILI biomarker to stratify at-risk patients for RILI toxicity for early intervention.

Malignant cancer cells use more than classic Warburg metabolism to achieve growth advantages and resistance. Many cancers instead upregulate both glycolysis and oxidative phosphorylation, creating a hybrid metabolic state. Metabolic hybridization defines a phenomenon where cancer cells simultaneously engage multiple energy pathways to adapt to treatment, environmental stress, or nutrient fluctuations (57). The hybrid metabolic phenotype often correlates with enhanced survival and metastatic potential and has been observed in melanoma, leukemia, gliomas, and endometrial carcinoma (57, 58). Our data suggest that radiation-induced myofibroblasts perform metabolic hybridization accelerating both glycolysis and oxidative phosphorylation. Overall, radiation induced FMT is a highly energetic process that increases the concentration of intra and extracellular lactate.

Although we previously reported that ionizing radiation promotes FMT in a lactate dependent manner (8), the source of lactate was not well characterized. This study is the first to implicate the PPP as a compensatory source of lactate and requirement for radiation-induced FMT and fibronectin deposition. This study revealed that G6PD and TKT are both potential novel therapeutic targets for RILI. Although polydatin or N3PT treatment in the mouse model of radiation injury was not explored in the present work, current evidence suggests inhibiting the PPP would have antifibrotic effects. For example, polydatin treatment reduced bleomycin-induced pulmonary fibrosis in mice (59) and protects mice from radon-induced radiation injury (60). N3PT is an analogue of thiamine (Vitamin B1) that competes with natural thiamine pyrophosphate as a stearic inhibitor for transketolase (42). Increased thiamine is associated with a 30% reduction in steatotic liver disease (61) and a reduction of cardiac fibrosis in a diabetic mouse model (62). Taken together, this supports the idea that thiamine, or N3PT, would also be antifibrotic in the RILI model. We also note that the coordinated mechanism of reverse PPP to drive forward glycolysis was not characterized. Our data suggest that radiation ignites a sophisticated coordination of metabolites involving both glucose and ribose. Taking a combinatorial therapeutic approach to target both G6PD and TKTL checkpoints may have utility for minimizing toxicity after radiation.

Although we do not know the exact source of the 5-carbon sugars that are incorporated into the PPP to act as an alternative energy source in irradiated fibroblasts, the most likely source in our cell culture experiments (which were performed in minimal essential medium containing glucose, except for Fig. 4) is catabolism of nucleic acids. During acute DNA repair and nucleotide degradation, intracellular ribose can derive from nucleotide salvage pathways from ribose-1-phosphate nucleoside degradation (63, 64). RNA degradation also releases ribose-containing nucleotides feeding into ribose-1-P and ribose-5-P pools (63, 65). In chronic injury states, autophagy-linked nucleotide recycling supplies nucleosides which can increase ribose-P (65). Regarding the mouse metabolomics results, the mouse diet used at our institution contains molasses which, as a source of fructose, can be converted to ribose (66).

Polydatin was originally identified as an inhibitor of G6PD activity (41) and has since been shown to be able to correct a wide variety of metabolic defects in metabolic disease models, including improving insulin resistance and glucose metabolism in models of diabetes and inhibiting proinflammatory and profibrotic growth factors in models of liver disease (67). Polydatin has multiple effects that might be antifibrotic (including modifying PI3K/Akt signaling (68)), and it is not clear whether these effects are all sequelae of blocking the PPP through G6PD or might be due to off target effects of polydatin. Given the strong links between the PPP and cancer proliferation, and the similarities between HLF and myofibroblasts in fibrosis and cancer cells (including the Warburg effect, (15)), our results support that the PPP is important in profibrotic metabolic reprogramming in HLFs.

Often, the PPP is thought to function to reduce oxidative damage and contribute to nucleotide repair. However, we are learning increasingly that the PPP is subject to change direction and priority based on environmental conditions and the needs of the cell (21, 65). Similarly, glycolysis is subject to the needs of the cell and can either prioritize precursor synthesis energy production or both (69). Ribose salvage to glycolysis occurs when glucose consumption by glycolysis decreases because it is prioritized to other metabolic pathways the cell needs to survive (65). Importantly, compensatory ribose salvage for energy has been previously reported in radioresistant cancer cells. In glioblastoma, cancer cells change their metabolism exactly as identified in radiation induced FMT. Radiation induces dimer PKM2, glucose-directed PPP activation, and PPP dependent lactate production. However, tetramerized PKM2 reduces glucose flux to the PPP, improves radiosensitivity, and decreases lactate production by “normalizing” glucose flux to a catabolic state (22). The redirection of glucose to meet synthetic demand and nonoxidative PPP induction allows ribose to serve as a carbon source for glycolysis which furthers pathologic metabolism.

Although we show a robust effect of targeting glycolysis and the PPP reducing radiation-induced FMT through reduced lactate production, this study has some limitations. Although targeting these pathways largely abrogates radiation induced FMT, we cannot rule out the possibility that other compensatory mechanisms may also promote pyruvate production. These pathways are only two of several metabolic pathways that are implicated in fibrometabolism (15). Because we used a targeted metabolomic approach that selected metabolites based on the untargeted metabolomic signatures identified in our pilot study of patients with RILI, we may have overlooked important pathways that were not part of the targeted panel. Also, our data does not provide spatial cell-specific metabolomic signatures. Emerging techniques such as spatial metabolomics will support cell-specific targeting for key players involved in fibrosis such as fibroblasts, epithelial cells, and resident inflammatory cells. However, our findings are similar to other previous reports of metabolic reprogramming in other RILI models and patients receiving radiation therapy. Our study did not include lung cancer in the model design or the influence of combinatorial therapy. Investigating the role of metabolism as a driver of radiation fibrosis in the presence of a tumor may improve translation of *in vivo* results to human patients.

In summary, this study is the first to establish a mechanism for how the PPP contributes to glycolysis and lactate production to enhance FMT. Importantly, we demonstrate that inhibition of glycolysis or the PPP restores normal lactate levels and inhibits myofibroblast differentiation, suggesting the potential use of PPP metabolism as a potential target in fibrosis treatment or metabolic biomarker for patient stratification and the selection of individualized treatment.

## Experimental procedures

All key reagents and resources are listed in the online Table S3.

### Radiation-induced pulmonary fibrosis mouse model

All animal experiments were performed with prior approval from the Virginia Commonwealth University Institutional Animal Care and Use Committee under the guidance of the Virginia Commonwealth University—Division of Molecular Radiobiology and Targeted Imaging departmental faculty. Male and female C57BL/6J mice aged 8 to 10 weeks (∼20 g female and ∼25 g male) were obtained from The Jackson Laboratory and acclimated for at least 7 days before irradiation. Mice were irradiated while under isoflurane anesthesia using a small animal radiation research platform (SARRP) irradiator (Xstrahl) for five consecutive days at 6 Gy per day, using a beam collimator of 1 cm × 1 cm that targeted the right lung while sparing the heart, liver, and spine as much as possible (Fig. S1). This model was chosen to replicate previous studies using a single 15 Gy thoracic dose (30), but using an updated, stereotactic fractionation approach.

BodyEquivalentDose=numberoffractions∗dose∗[1+(dose/α/β)]

Lunglateeffects,α/β=3Gy

FractionateddoseBED=5∗6∗[1+(6/3)]=90Gy

SingledoseBED=1∗15∗[1+(15/3)]=90Gy

The targeted location was determined using low-dose CT imaging per mouse immediately before radiation treatment delivery. Mice from the control groups were anesthetized but did not receive radiation (Fig. S1).

Mice were euthanized 14 days (acute inflammatory phase) and 180 days (fibrotic phase) post irradiation. The lungs were perfused by injecting 1× PBS into the right side of the heart, and then removed from the thoracic cavity. The lungs were lavaged twice with 0.5 ml 1× phosphate buffered saline (PBS), which was collected and saved. The right (irradiated) lobe was separated into the upper, middle, and lower lobes for subsequent analysis. The upper lobe was further cut in half and utilized to extract RNA or protein, the middle lobe was fixed in formalin and embedded in paraffin for immunohistochemistry analyses, the bottom lobe was used for LC/MS and targeted metabolomics. The BAL was centrifuged for 5 min at 5000*g* and the BAL fluid was frozen at −80 °C. The cell pellet was resuspended in PBS, counted with a hemacytometer, and 50,000 cells were applied to glass slides using a cytospin centrifuge (Thermo Fisher Scientific). BAL cells were stained with Epredia Richard-Allan Scientific Three-Step Stain Kit (Thermo Fisher Scientific), and differential cell counts were performed.

### Primary HLF radiation model

Primary HLF strains were derived from nonfibrotic donors and cultured using the explant technique as previously described (70). Fibroblasts were positive for vimentin and negative for CD45, cytokeratin, and CD34. Donors gave informed consent, and the protocol was approved by Virginia Commonwealth University’s (VCU’s) Institutional Review Board, and conforms to the Declaration of Helsinki principles. HLFs were used at passage 5 to 7, see Table S2 for further donor information. Fibroblasts were cultured in minimum essential media (Thermo Fisher Scientific) supplemented with 10% fetal bovine serum (Sigma-Aldrich), 2 mM L-glutamine (Thermo Fisher Scientific, and 1% antibiotic-antimycotic (GIBCO) at 37 °C with 7% CO_2_. Medium was changed immediately prior to irradiation and then 3 days post irradiation. For irradiation experiments, HLFs were seeded at ∼10,000 cells/cm^2^ in either 6-well, 24-well, or 96-well dishes, depending on the experiment. Because irradiation slows cell division, control (nonirradiated) wells were seeded at half the usual density so they would not overgrow during the experiment. HLFs were irradiated using an X-ray source (CIXD Dual Headed Irradiator, Xstrahl) at a dose rate of 1.28 Gy per minute which is a clinically verified output. Unless otherwise indicated, HLFs were harvested 5 days after irradiation.

### Lung tissue histological staining

The right middle lung lobe was fixed with 10% neutral buffered formalin (VWR) and processed for histological analysis. Hematoxylin & Eosin staining was performed on 5-μM thick tissue sections, and staining for collagen fibers was performed using Trichrome Stain AB solution (Sigma-Aldrich).

### Preparation of mouse lung tissue for LC-MS/MS system

Lung homogenates were analyzed at the VCU Lipidomics and Metabolomics Shared Resource. The lower right lobes of lung tissue from the irradiated or control mice were promptly frozen in liquid nitrogen then stored at −80 °C before extraction. Frozen tissues were weighed to determine wet lung mass before being homogenized into 874 μl cold 80% methanol (ACS grade, Colonial Scientific) with an OMNI-TH01 homogenizer (OMNI International). After cooling samples on ice briefly, 540 μl ice cold chloroform was added followed by a second homogenization. Subsequently, 360 μl of ice-cold chloroform and 360 μl ice-cold ultra-pure water were added into the homogenized samples to separate polar and nonpolar fractions. The mixture was vortexed and allowed to sit for 10 to 15 min on ice. After centrifugation at 4 °C for 10 min at 5000 rpm, the supernatant was transferred to a clean tube and dried in a Speed-Vac concentrator (SPD 2010 Savant, Thermo Fisher Scientific) and stored at −80 °C freezer until analysis. Before loading on LC-MS, the dried extract was reconstituted with 100 to 300 μl water based on the weight of lung tissue to reduce effect of matrix concentration. Five microliters reconstituted samples were injected into LC-MS. The results were normalized with tissue weight.

### Preparation of primary HLFs for LC-MS/MS system

HLFs in six well dishes were irradiated and cultured for 5 days as described above. The HLFs were washed twice with warm (37 °C) 1x PBS before quenching the plate by setting it in a dry ice-ethanol bath. The frozen plate was thawed on wet ice, and cells were collected by scraping with 1 ml 80% cold methanol, which was then transferred to microcentrifuge tubes and lysed with sonication. The cell lysates were vortexed for 10 min at 4 °C to extract metabolites, and the supernatants were collected after centrifuging at 10,000 rpm at 4 °C for 10 min. An additional 0.2 ml of 80% methanol was added to the precipitate followed by agitating, vortex mixing, and centrifugation to further extract metabolites. The supernatant from both extractions was combined and dried in a Speed-Vac concentrator (SPD 2010 Savant, Thermo Fisher Scientific). The dry extract was stored in −80 °C freezer until analysis. Before analysis, the extract was reconstituted with 100 μl H_2_O and centrifuged. Five microliters was injected into the liquid chromatography-tandem mass spectrometry (LC-MS/MS) system. The pellet was reconstituted in 1x radioimmunoprecipitation assay (RIPA) solution before analyzing for total protein content by bicinchoninic acid (BCA) assay.

### LC-MS/MS measurement

All LC-MS/MS analyses were performed on an AB SCIEX (AB SCIEX LLC) Q Exactive Orbitrap instrument TRAP 6500 system (Thermo Fisher Scientific), which consists of a SHIMADZU Nexera ultra high-performance liquid chromatography system coupled with a hybrid triple quadrupole and ion trap mass spectrometer. Analyst 1.6 software was used for system control and data acquisition, and MultiQuant 3.0 software was used for data processing and quantitation.

A total of 89 metabolite standards were used for calibration standard curve. All these reference standards were purchased from Sigma-Aldrich and Thermo Fisher Scientific with minimum of 95% purity or the highest available purity. Solvents used for metabolites extraction and liquid chromatography including hexafluora-2-isopropanol (HFLP), triethylamine (TEA), formic acid, acetonitrile, methanol, and water were LC-MS grade from Thermo Fisher Scientific. Selected metabolites across glycolysis, TCA cycle and PPP were separated with two methods of reverse-phase LC and ion-pairing LC for the best retention on chromatograph (71). Thermo Accucore Vanquish C18+ column (2.1 mm × 150 mm, 1.5 μm) was used for reverse phase separation to separate saccharides taken up by cells and small organic acids in TCA pathways with the mobile phase A of 0.1% formic acid in water and B of 0.1% formic acid in acetonitrile at flow rate of 0.2 ml/min at 40 °C. The LC gradient started from 0% B for 0.5 min, increased to 46% B in 10 min, ramped to 100% B at 10.5 min, after holding 1.5 min and decreased to 0% B at 12.1 min.

Ion-paring method was applied to separate phosphates-based metabolites on glycolysis and PPP with an Atlantis T3 column (2.1 mm × 100 mm, 3.0 μm) at the flow rate of 0.5 ml/min at 30 °C. Briefly, 100 mM hexafluoroisopropanol (HFIP) and 8.6 mM triethylamine in water (final pH, 8.3 ± 0.1) was optimized as mobile phase A, and 10% acetonitrile in mobile phase A was used as mobile phase B. LC gradients started at 0% B for 0.5 min, reached 10% B at 8 min, ramped to 100% B at 13 min, held for 1 min and dropped to initial 0% B at 14.1 min. A quality control of 1 μM standard mixture was used to monitor the intensity variation of every 10 injections of samples.

The mass spectrometric parameters of ionization polarity, product ion, collision energy, decluttering potential, and cell exit potential of all metabolites were manually tuned and optimized for best sensitivity by direct infusion. Multiple reaction monitoring mode with above optimal parameters were used for analysis with ion spray potential of 5500 V for positive mode and 4500 V for negative mode with nebulizer gas (GS1) and bath gas (GS2) at 40 psi and 25 psi respectively. Curtain gas (CUR) was 30 psi, and collision gas (CAD) was set to medium level; source temperature (TEM) was 450 °C. The dwell time for each transition was optimized ranging from 4 ms to 80 ms. The transition and detailed mass spectrometric parameters are listed in Table S4.

### Western blotting

Primary human lung fibroblasts were seeded and irradiated as described. A 70% media change was performed on day 3, and cell lysates were harvested for western blot on day 5. Briefly, the wells were washed with warm 1x PBS, then the cells were lysed by scraping with RIPA buffer (Cell Signaling Technologies, 9806S) supplemented with protease inhibitor cocktail and 1 mmol/L phenylmethylsulfonyl fluoride (Sigma-Aldrich, 10837091001). Lysates were then sonicated followed by centrifugation to pellet any cellular debris. Protein concentration was determined by the bicinchoninic acid assay (Thermo Fisher Scientific). Cell lysates containing 2 to 5 μg protein were loaded per lane and separated by 10% SDS-PAGE and transferred to polyvinylidene difluoride membrane, and proteins of interest were detected by western blot analysis and chemiluminescence. Before blocking, total protein and transfer efficiency were detected by Ponceau stain (Thermo Fisher Scientific) according to the manufacturer’s protocol. Blots were blocked and incubated with antibodies in 5% nonfat dry milk in 1x PBS plus 0.05% Tween-20. Antibodies are listed in Table S3.) Lactate dehydrogenase A (Cell Signaling Technology; 2012S 1:1000) was detected overnight at 4 °C. α-Smooth Muscle Actin (Sigma-Aldrich, A2547 1:40,000), Fibronectin (Sigma-Aldrich, F3648 1:5000), and β-Tubulin (Abcam; ab6046 1:20,000) were incubated for 1 h at room temperature. Secondary antibody was goat anti-rabbit (Jackson ImmunoResearch, 115-035-144 1:10,000) and goat anti-mouse (Jackson ImmunoResearch; 115-035-146 1:10,000) incubated for 1 h at room temperature on a rocker. Blots were developed with Immobilon Western Chemiluminescent HRP Substrate (Thermo Fisher Scientific) and imaged using a C-Digit scanner (LI-COR Biosciences). Quantitation was performed using Image Studio Version 5.5 (LI-COR Biosciences). Results are expressed as normalized to the loading control (β-Tubulin).

### Immunofluorescence staining

Primary human lung fibroblasts were irradiated in 96-well optical-bottom microplates (Thermo Fisher Scientific) followed by a 70% media change on day 3. On day 5, the cells were washed three times with warmed PBS and fixed in 4% paraformaldehyde for 15 min. Wells were washed with PBS and blocked with 5% normal goat serum (Jackson ImmunoResearch) in 1x PBS for 1 h, followed by α-Smooth Muscle Actin (Sigma Aldrich; A2547, 1:400) in blocking solution overnight at 4 °C. The next day, wells were washed with PBS and incubated with Fibronectin-AF488 conjugate (Abcam; ab198933 1:400) in blocking solution for 90 min at room temperature on a rocker covered from light. The wells were washed with PBS, and αSMA was detected with goat anti-mouse-AF555 (Southern Biotech, 1032-30 1:500) in blocking solution for 60 min at room temperature on a rocker covered from light. Cells were washed for a final time, counterstained with 4′,6-diamidino-2-phenylindole (DAPI) (Thermo Fisher Scientific) to visualize nuclei and imaged using an EVOS M7000 microscope (Thermo Fisher Scientific) and analyzed with Celleste Software 6.0.

### Seahorse bioanalyzer

Primary human lung fibroblasts were cultured on XF24 well plates (Agilent Technologies) and irradiated at the indicated doses. Cells were seeded at 12,500 cells per well (n = 10) 48 h before irradiation (0, 3 or 5 Gy). Five days after irradiation, glycolysis was measured using the Seahorse XF Glycolytic Rate Assay kit (Agilent Technologies) following the manufacturer’s instructions. Final concentrations of rotenone and antimycin A were 0.5 μM each, and the final concentration of 2-deoxyglucose was 50 mM. Mitochondrial respiration was analyzed using the Seahorse XF Cell Mito Stress Test (Agilent Technologies) as instructed; final concentrations of Oligomycin were 2 μM, FCCP 2.3 μM, and rotenone/antimycin A 0.5 μM each. Data were normalized to cell count by Hoechst stain (2 μM) using a Lionheart FX automated microscope (Agilent Technologies).

### Extracellular lactate measurements

Lactate was measured in cell supernatants using a Lactate Colorimetric/Fluorometric Assay Kit (Abcam; ab6533) according to company specifications with one exception. Samples were prediluted 1:1 to fall within the allotted standard curve. Absorbance was measured on an iMark microplate absorbance reader (Bio-Rad Laboratories).

### Compound and RNA inhibition

All inhibitor compounds were purchased from Medchem Express as shown in Table S3. For inhibition of selected metabolic enzymes, Compound 3K (1.0 μM, inhibits PKM2), polydatin (1.0 μM, inhibits glucose-6-phosphate-dehydrogenase), N3PT (0.5 μM, inhibits transketolase) or shikonin (0.5 μM, inhibits PKM2) were added to HLF cultures 1 h before irradiation. Medium was changed on day 3, and the compounds were replaced at the same concentration. HLFs were harvested and analyzed on day 5. All compounds were prepared as stock solutions in dimethyl sulfoxide (DMSO), and DMSO (0.02%) was added to negative control wells. To genetically inhibit PKM2, HLFs were transfected 48 h before irradiation with either a Smart Pool ON-TARGET siRNA or Smart Pool Non-Targeting Control Pool (Dharmacon) using Lipofectamine RNAiMAX transfection reagent (Thermo Fisher Scientific). Fibroblasts were cultured in minimum essential media (Thermo Fisher Scientific) supplemented with 10% fetal bovine serum (Sigma-Aldrich), 2 mM L-glutamine, with no antibiotic-antimycotic added at 37 °C with 7% CO_2_. Medium was changed immediately prior to irradiation and then after every 3 days post irradiation. Medium was left antibiotic-antimycotic free starting on the day of transfection and for the remainder of the experiment. For irradiation experiments, HLFs were seeded at ∼10,000 cells/cm^2^ in either 6-well, 24-well or 96-well dishes, depending on the experiment. A 100 μl master mix containing the siRNA—lipofectamine complex was made prior to adding to the cells. The lipofectamine and siRNA were prepared in separate Eppendorf tubes before combining to make up the final 100 μl master mix. Lipofectamine RNAiMax was diluted in Opti-MEM (Thermo Fisher Scientific) such that the dilution was 3%, and the siRNA was diluted in Opti-MEM (Thermo Fisher Scientific) such that the final concentration was 25 pmol per well. Both solutions were scaled up to account for the number of technical replicates for the experiment. After combining, the master mix was incubated at room temperature for 5 min before adding 100 μl per well.

### Transketolase assay

HLFs were seeded and irradiated as described and cultured for 5 days. One hour prior to harvest, N3PT (0.05 μM or 0.1 μM) in DMSO or DMSO vehicle was added. Cells were lysed, and a transketolase assay was performed on the lysates using a commercial TKT activity assay kit (Abcam, ab273310).

### Statistical analysis

All data are expressed as ± SD. For comparisons of two groups with one variable, statistical significance was determined using a two-tailed, unpaired Student’s *t* test. For comparison of multiple treatment groups, One-way or two-way ANOVA with Dunnett’s multiple comparison post test was used where indicated. A *p* value ≤ 0.05 was considered statistically significant. All statistical analysis was conducted using GraphPad Prism (versions 10.5.0 through 11.0, GraphPad, La Jolla, CA). Principal component analysis of metabolomics results from irradiated mouse lung tissue (Fig. 1*B*) was performed using MetaboAnalyst 5.0.

## Data availability

The raw targeted metabolomics results and mass spectrometry transitions for each metabolite measured are presented in the Supplement.

## Ethical standards

The manuscript does not contain clinical studies or patient data. Primary human cell strains were derived from donor tissue. Donors gave informed consent and the protocol was approved by the Virginia Commonwealth University Institutional Review Board (protocol number HM20017903). Mouse experiments were performed under the supervision of the Virginia Commonwealth University Institutional Animal Care and Use Committee (protocol number AD10002138).

## Supporting information

This article contains supporting information including Tables S1–S4 and Figures S1–S4, and metabolomic data for Figures 1, 3, 4 and 5.

## Conflict of interest

PJS has participated in clinical trials with ROCHE Genetech, and Bristol Myers Squibb. She is a shareholder in Galecto (self) and Permeaderm (spouse). She is on the board of the American Thoracic Society and is on the editorial board for the American Journal of Respiratory and Critical Care Medicine. She has previously received consulting fees from companies with an interest in pulmonary fibrosis, including Boehringer Ingelheim, UCB Pharma, Fibrogen, VYNE, Avalyn Pharma and Three Lakes Foundation; has reviewed grants for Boehringer Ingelheim and the Parker B. Francis Foundation; and has received travel reimbursement from the Pulmonary Fibrosis Foundation and the American Thoracic Society. MATF has received travel reimbursement from the Pulmonary Fibrosis Foundation. PJS, MATF and THT have previously received funding from the NIH, Novomedix and UCB Pharma for research unrelated to this work. PJS, MATF and THT hold intellectual property related to fibrosis. The other authors have no financial interests to disclose.
