# Supplementary material for: The pentose phosphate pathway contributes to excess lactate production in radiation-induced fibroblast to myofibroblast transdifferentiation
Source: J Biol Chem. 2026 May 13;302(7):113142. doi: 10.1016/j.jbc.2026.113142 (PMC13276329; doi:10.1016/j.jbc.2026.113142)
Supplement: Supporting Figures and Tables [file mmc2.pdf]

Supplemental Table S1. Metabolites included in the targeted metabolomic analysis with their associated KEGG pathway assignments.

Metabolite	Pathway	KEGG
**3-Phosphoglyceric acid**	Glycolysis	C00597
**Fructose-1,6-2P**	Glycolysis	C00354
**Glucose-6-P**	Glycolysis	C00668
**Glyceraldehyde-3-P**	Glycolysis	C00118
**Phosphoenolpyruvate**	Glycolysis	C00074
**Glucose**	Glycolysis	C00031
**Lactate**	Glycolysis	C00186
**Pyruvate**	Glycolysis	C00022
**Methionine**	Methionine Metabolism	C00073
**Nicotinic acid**	Nicotinic/Nicotinamide Metabolism	C00253
**Nicotinamide**	Nicotinic/Nicotinamide Metabolism	C00153
**Erythrose-4-P**	Pentose Phosphate Pathway	C00279
**Ribose-5-P**	Pentose Phosphate Pathway	C00117
**Sedoheptulose-7-P**	Pentose Phosphate Pathway	C02076
**Glutathione**	Pentose Phosphate Pathway	C00051
**GTP**	Purine metabolism	C00044
**UTP**	Purine metabolism	C00075
**ADP**	Purine/Pyrimidine Metabolism	C00008
**AMP**	Purine/Pyrimidine Metabolism	C00020
**ATP**	Purine/Pyrimidine Metabolism	C00002
**Adenine**	Purine/Pyrimidine Metabolism	C00147
**Cysteine**	Purine/Pyrimidine Metabolism	C00097
**Guanine**	Purine/Pyrimidine Metabolism	C00242
**IMP**	Purine/Pyrimidine Metabolism	C00130
**Uridine**	Purine/Pyrimidine Metabolism	C00299
**Cytidine**	Purine/Pyrimidine Metabolism	C00475
**Acetyl-CoA**	Tricarboxylic Acid Cycle	C00024
**Malonyl-CoA**	Tricarboxylic Acid Cycle	C00083
**Succinyl-CoA**	Tricarboxylic Acid Cycle	C00091
**Aconitic acid**	Tricarboxylic Acid Cycle	C00417
**Alpha-KG**	Tricarboxylic Acid Cycle	C00026
**Aspartic acid**	Tricarboxylic Acid Cycle	C00049
**Citrate**	Tricarboxylic Acid Cycle	C00158
**Fumarate**	Tricarboxylic Acid Cycle	C00122
**Malate**	Tricarboxylic Acid Cycle	C00149
**Maleic acid**	Tricarboxylic Acid Cycle	C01384
**Succinate**	Tricarboxylic Acid Cycle	C00042
**Phenylalanine**	Tyrosine Metabolism	C00079

**Alanine**	Amino Acid Metabolism	C00041
**Arginine**	Amino Acid Metabolism	C00062
**Asparagine**	Amino Acid Metabolism	C00152
**Histidine**	Amino Acid Metabolism	C00135
**Isoleucine**	Amino Acid Metabolism	C00407
**Leucine**	Amino Acid Metabolism	C00123
**Serine**	Amino Acid Metabolism	C00065
**Thymidine**	Amino Acid Metabolism	C00214
**Tryptophan**	Amino Acid Metabolism	C00078
**Tyrosine**	Amino Acid Metabolism	C00082
**Valine**	Amino Acid Metabolism	C00183
**Carnitine**	Carnitine Shuttle	C00318
**Palmitoyl-carnitine**	Carnitine Shuttle	C02990
**Acetoacetyl-CoA**	Fatty Acid β-Oxidation	C00332
**CoA**	Fatty Acid β-Oxidation	C00010
**Glutaryl-CoA**	Fatty Acid β-Oxidation	C00527
**Palmitoyl-CoA**	Fatty Acid β-Oxidation	C00154
**Glutaric acid**	Fatty Acid β-Oxidation	C00489
**Malonic acid**	Fatty Acid β-Oxidation	C00383
**Palmitic acid**	Fatty Acid β-Oxidation	C00249
**GDP-Mannose**	Fructose-Mannose Metabolism	C00096
**6-phosphogluconate**	Gluconic Shunt Pathway	EC 1.1.1.44
**Gluconic acid**	Gluconic Shunt Pathway	C00257
**Glutamate**	Glutamate Metabolism	C00025
**Glutamine**	Glutamine Metabolism	C00064
**Proline**	Glutamine Metabolism	C00148
**Glycine**	Glycine, Serine, Threonine Metabolism	C00037
**Ribose**	Glycine, Serine, Threonine Metabolism	C00121
**Threonine**	Glycine, Serine, Threonine Metabolism	C00188

Supplemental Table S2. Donor characteristics for primary human fibroblast strains

Donor	Age	Sex	Diagnosis (reason for biopsy)	Figures used in
**1**	60	F	Adenocarcinoma	2,3,6,7
**2**	68	M	Subpleural Fibro elastotic changes	2
**3**	54	F	Adenocarcinoma	2,4,6,7
**4**	53	M	Squamous Cell Carcinoma	2,3,5
**5**	67	F	Adenocarcinoma	4
**6**	70	F	Adenocarcinoma	3

Supplemental Table S3. Key reagents and resources.

Antibodies	Company Source & Location	Identifier	kDa
alpha-Smooth muscle actin (αSMA) (1:40,000)	Sigma-Aldrich (Burlington, MA, USA)	A2547	42
beta-Tubulin (βTUB) (1:20,000)	Abcam (Cambridge, UK)	ab6046	50
BLUEstain™ Protein ladder, 11-245 kDa	Gold Biotechnology (St. Louis, MO, USA)	P007-500	
Goat – anti-mouse - AF555 (1:500)	Southern Biotechnology (Birmingham, AL, USA)	103632	
Fibronectin - AF488 (GFP) (1:500)	Thermo Fisher Scientific (Waltham, MA, USA)	53986982	
Fibronectin (FN) (1:5,000)	Sigma-Aldrich (Burlington, MA, USA)	F3648	220
Lactate dehydrogenase A (LDHA) (1:10,000)	Cell Signaling Technology (Danvers, MA, USA)	2012S	36
Peroxidase AffiniPure Goat Anti-Mouse IgG (H+L) (1:10,000)	Jackson ImmunoResearch (Philadelphia, PA, USA)	115-035-146	
Peroxidase AffiniPure Goat Anti-Rabbit IgG (H+L) (1:10,000)	Jackson ImmunoResearch (Philadelphia, PA, USA)	111-035-144	
Protein Standard Precision Plus	Bio-Rad Laboratories (Hercules, CA, USA)	161-0376	
Pyruvate kinase m2 (PKM2)	Cell Signaling Technology (Danvers, MA, USA)	4053T	60
StrepTactin-Horseradish Peroxidase (HRP) *for chemi Ladder*	Bio-Rad Laboratories Inc. (Hercules, CA, USA)	1610381	

Chemicals	Source	Identifier	
5X siRNA Buffer	Horizon Discovery Dharmacon	B-002000-UB-100	
Agilent Technologies XF BASE MEDIUM 2X1L	Agilent Technologies	102353-100	
Ammonium persulfate	Bio-Rad Laboratories (Hercules, CA, USA)	1610700	
Iscript™ Reverse Transcription Supermix	Bio-Rad Laboratories (Hercules, CA, USA)	1708841	
Lipofectamine RNAiMAX Transfection Reagent—In Vitro Delivery of siRNA	Thermo Fisher Scientific (Waltham, MA, USA)	13778100	
Minimum Essential Medium	Thermo Fisher Scientific (Waltham, MA, USA)	11095098	
Normal Goat Serum	Jackson ImmunoResearch	005-000-121	
RIPA Lysis Buffer, 10X	Sigma-Aldrich (Burlington, MA, USA)	20-188	
Opti-MEM™ I Reduced-Serum Medium	Thermo Fisher Scientific (Waltham, MA, USA)	31985062	
Paraformaldehyde 20% solution, EM Grade	Electron Microscopy Sciences (Hatfield, PA, USA)	15713-S	
ProtoGel 30%	National Diagnostic (Atlanta, GA, USA)	EC890450ML	
Iq™ Multiplex Powermix	Bio-Rad Laboratories (Hercules, CA, USA)	1725848	
Compound 3K	Medchem Express (Monmouth Junction, NJ, USA)	HY-103617	
Polydatin	Medchem Express (Monmouth Junction, NJ, USA)	HY-N0120A	
Shikonin	Medchem Express (Monmouth Junction, NJ, USA)	HY-N0822	
N3-pyridyl thiamine	Medchem Express (Monmouth Junction, NJ, USA)	HY-16339	
Trichrome Stain AB Solution	Sigma-Aldrich (Burlington, MA, USA)	HT10516	

Critical commercial assays	Source	Identifier	
BCA Protein Assay	Thermo Fisher Scientific (Waltham, MA, USA)	23225	
Epredia™ Richard-Allan Scientific™ Three-Step Stain Kit	Thermo Fisher Scientific (Waltham, MA, USA)		
Lactate Colorimetric/Fluorometric Assay Kit	Abcam (Cambridge, United Kingdom)	ab65330	
Prestoblue™ Cell Viability Reagent	Thermo Fisher Scientific (Waltham, MA, USA)	A13262	
QIAwave RNA Plus Mini Kit	Qiagen (Hilden, Germany)	74634	
Seahorse XF Glycolytic Rate Assay Kit	Agilent Technologies (Santa Clara, CA, USA)	103344-100	
Seahorse XF Cell Mito Stress Test Kit	Agilent Technologies (Santa Clara, CA, USA)	103015-100	
Seahorse XF MitoFuel Flex Test Kit	Agilent Technologies (Santa Clara, CA, USA)	103270-100	
**Oligonucleotides**	**Source**	**Identifier**	
ON-TARGET plus Non-targeting Pool	Horizon Discovery Dharmacon (Lafayette, CO, USA)	D-001810-10-05	
ON-TARGET plus Human PKM2 siRNA (5315)	Horizon Discovery Dharmacon (Lafayette, CO, USA)	L-006781-00-0005	
**Additional Compounds**			
D(-)-Ribose, 99+%	Thermo Fisher Scientific (Waltham, MA, USA)	CAS: 50-69-1	
D-Ribose (U-$^{13}C_5$, 98%)	Cambridge Isotope Laboratories (Tewksbury, MA, USA)	CAS: 202114-47-4	
D-(+)-Glucose, anhydrous, 99%	Thermo Fisher Scientific (Waltham, MA, USA)	CAS: 50-99-7	
D-Glucose (U-$^{13}C_6$, 99%)	Cambridge Isotope Laboratories (Tewksbury, MA, USA)	CAS:110187-42-3	
Glucose-free DMEM	Thermo Fisher Scientific (Waltham, MA, USA)	A1443001	

Supplemental Table S4. Mass Spectrometry Transition Table.

Metabolite	Mode	Q1	Q3	DP	CE	CXP
Adenine	Pos	135.7	119	95	30	10
Adenosine	Pos	268.7	136	60	20	12
alanine	Pos	89.8	44.2	25	20	7
Allantion	Pos	159.3	116	47	8.6	10
Arginine	Pos	174.9	70.3	40	30	6
Asparagine	Pos	132.9	87.3	40	12	12
Asptartic acid	Pos	133.9	74.3	40	15	10
carnitine	Pos	162.3	103.2	35	23	11
cyclic_AMP	Pos	329.6	136.2	37	37	10
cysteine	Pos	121.8	76.3	25	18	5
cystine	Pos	241	74.2	35	35	10
cytidine	Pos	244	112.2	15	17	10
Glutamate	Pos	148	84.2	60	20	9
Glutamine	Pos	147	84.3	30	26	12
Gluthionine	Pos	307.7	179.3	30	19	11
glycine	Pos	75.7	30.3	30	18	14
Guanine	Pos	151.7	135	90	25	12
Guanosine	Pos	284.3	152	50	15	10
histidine	Pos	155.8	110.2	50	19	7
hypoxathine	Pos	137	92	135	35	10
IMP	Pos	349.2	137	55	15	8
Inosine	Pos	269.3	137	50	14	7
iso-leucine	Pos	132	69.3	50	28	10
leucine	Pos	131.9	86.3	20	15	7
lysine	Pos	146.7	84.3	25	18	15
Methionine	Pos	149.9	104.3	30	14	8
nicotinamide	Pos	123	80.2	40	27	9
nicotinic acid	Pos	124	80.2	65	28	11
palmitoyl-carnitine	Pos	400	341.3	35	26	29
phenylalanine	Pos	165.9	120.3	40	21	8
proline	Pos	116.1	70.3	35	25	30
purine	Pos	121	94	47	33	10
pyrimidine	Pos	81	54	50	35	15
serine	Pos	106.2	60.2	45	15	5
threonine	Pos	119.7	57.4	30	18	10
thymidine	Pos	243	127.2	30	13	12
tryptophan	Pos	205.1	188.2	35	15.3	13.5
tyrosine	Pos	182	136.3	110	19	5
Urea	Pos	60.7	44.3	70	25	5
uridine	Pos	245	113.2	15	14	11
valine	Pos	117.8	55.3	25	25	9
Xanthine	Pos	153	110	78	24	12
2HG	Neg	146.9	128.9	-15	-10	-10

3-Phosphoglyceric acid	Neg	185	97	-15	-23	-11
6-phosphogluconate	Neg	275	97	-30	-22.8	-10
acetoacetate	Neg	100.9	57	-13	-14	-7
Acetoacetyl-CoA	Neg	850	766	-95	-38	-25
Acetyl-CoA	Neg	808.1	408.1	-130	-46	-18
aconitic acid	Neg	172.8	129	-11	-13	-9
ADP	Neg	426	327.8	-65	-24	-22
alpha-KG	Neg	145	100.9	-32	-10	-13
AMP	Neg	346	78.8	-130	-70	-10
ATP	Neg	506.1	272.9	-110	-37	-11
beta-hydroxybutyrate	Neg	103	59	-15	-12	-9
Citrate	Neg	190.9	111	-30	-20	-15
CoA	Neg	766	407.8	-155	-43	-35
CTP	Neg	482	384	-125	-25.5	-16
Erythrose-4-P	Neg	199	97	-40	-14	-13
Fructose-1,6-2P	Neg	339	97	-70	-26	-8
furmarate	Neg	115	71	-25	-10	-7
GDP-Mannose	Neg	604	424	-16	-40	-22
gluconic acid	Neg	194.9	129	-40	-18	-13
glucose	Neg	178.8	89	-80	-14	-10
Glucose-6-P	Neg	258.9	97	-120	-16	-10
glutaric acid	Neg	131	87	-30	-14	-7
Glutaryl-CoA	Neg	880	408	-160	-54	-32
Glyceraldehyde-3-P	Neg	169	151	-93	-8	-5
GMP	Neg	361.7	79	-50	-85	-9.5
GTP	Neg	522.1	423.9	-120	-27	-12
iso_citric acid	Neg	190.8	72.9	-30	-15	-20
itaconic acid	Neg	128.9	84.9	-20	-14	-11
lactate	Neg	88.8	43	-63	-15	-7
Malate	Neg	132.8	114.8	-45	-16	-10
maleic acid	Neg	115.1	71.1	-11	-14	-8
malonic acid	Neg	102.9	59	-7	-12	-7
malonyl-CoA	Neg	851.8	808	-77	-34	-24
NAD	Neg	662	540	-135	-23	-16
NADH	Neg	664.2	408	-115	-38	-15
NADP	Neg	742.2	620	-75	-20	-25
palmitic acid	Neg	255	237	-130	-27.8	-16
palmitoyl-CoA	Neg	1004.5	408	-150	-55	-22
Phos-pyruvate	Neg	167	79	-53	-15	-8
pyruvate	Neg	86.9	43	-11	-11	-6
Ribose	Neg	149	89	-25	-8	-9
Ribose-5-P	Neg	229.1	97	-100	-14	-8
Sedoheptulose-7-P	Neg	289	97	-30	-21	-11
succinate	Neg	116.8	72.9	-30	-15	-6
Succinyl-CoA	Neg	866.1	408.2	-150	-52.5	-17
taurine	Neg	123.8	80	-9	-30	-13
UMP	Neg	323	211	-100	-21	-11
UTP	Neg	482.9	384.9	-70	-24	-30

Supplemental Figure S1

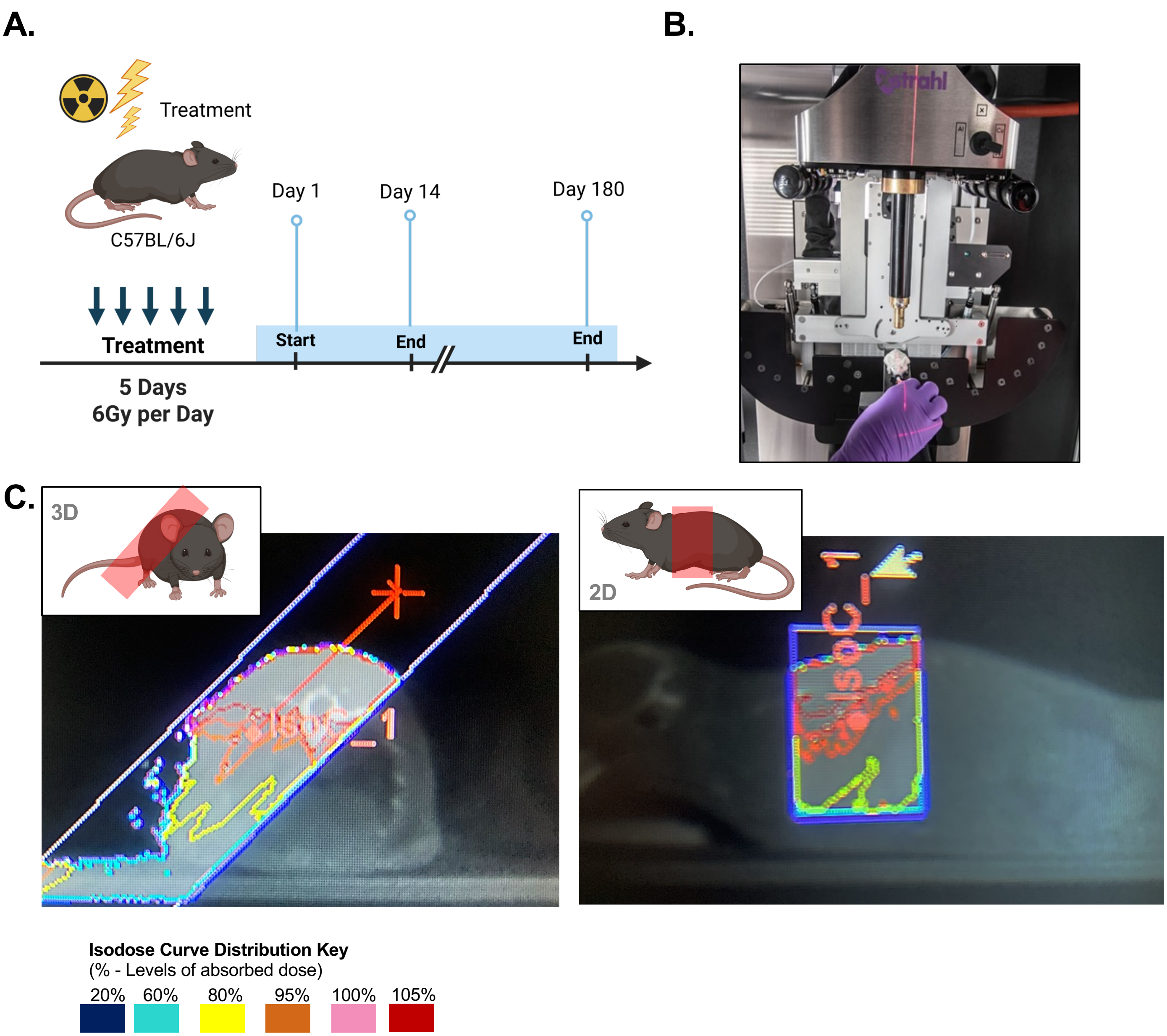

Supplemental Figure 1. Hypofractionated Stereotactic micro-CT guided targeted thoracic radiation model. A. Experimental design of the mouse irradiation model. (Created with biorender.com). **B.** Mouse placement on stage using the collimated 1 cm x 1cm system. **C.** Example micro-CT of mouse from a 3D and 2D view pre-determined before delivering radiation treatment dose. Isocurve distribution listed accordingly.

Supplemental Figure S2

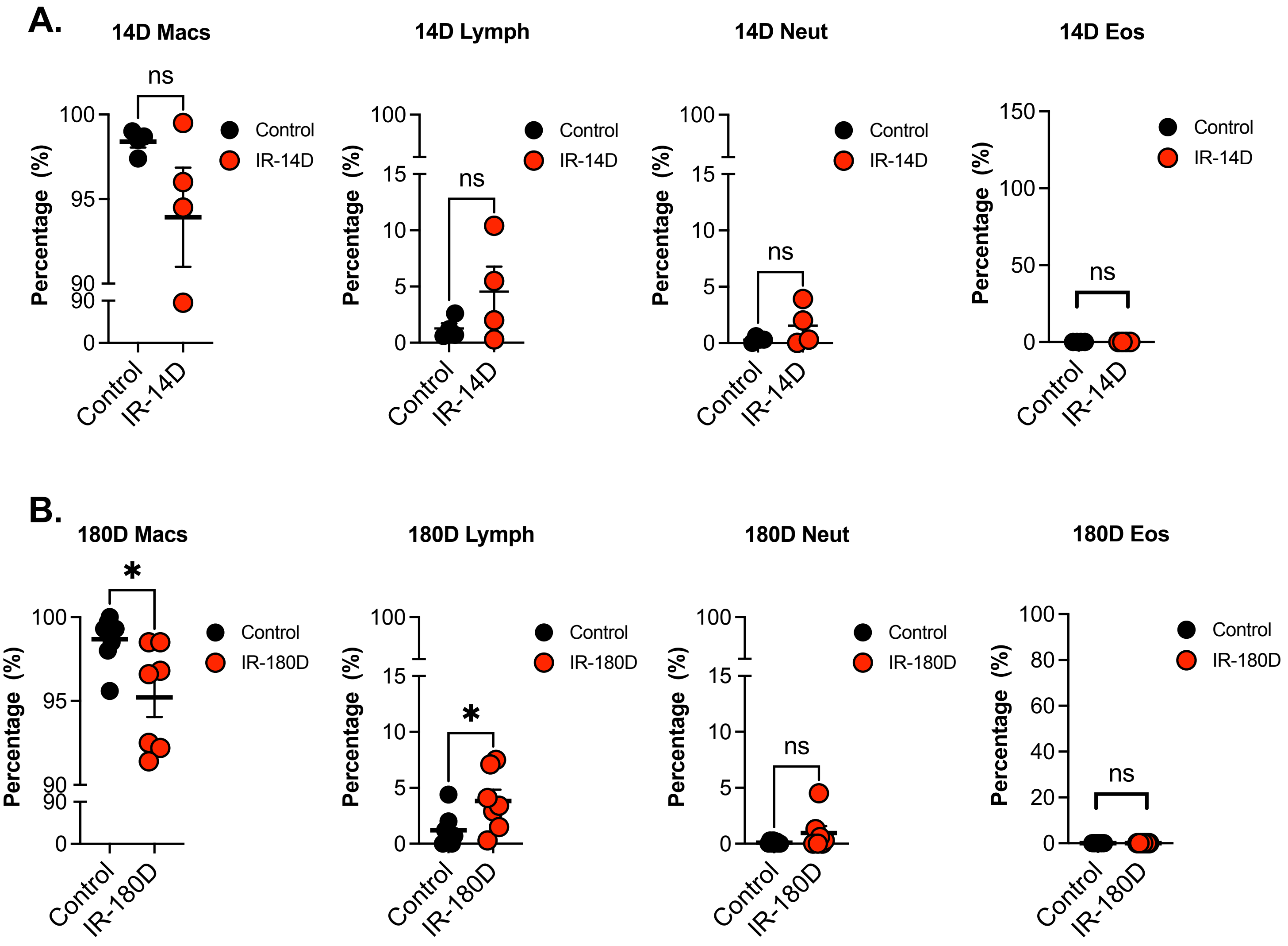

Supplemental Figure 2. Inflammatory cell populations in the bronchoalveolar lavage fluid in the mouse irradiation model. Groups of mice were irradiated as described and harvested as described. BAL cells were counted, applied to glass slides with a Cytospin centrifuges, stained and differential counts were performed. **A.** BAL cell percentages were determined at 14 days after irradiation. **B.** BAL cell percentages were determined at 180 days after irradiation. IR, irradiated; Macs, macrophages; Lymph, lymphocytes; Neut, neutrophils; Eos, eosinophils. Significance was assessed by 2-tailed t-test, * $P \leq 0.05$.

Supplemental Figure S3

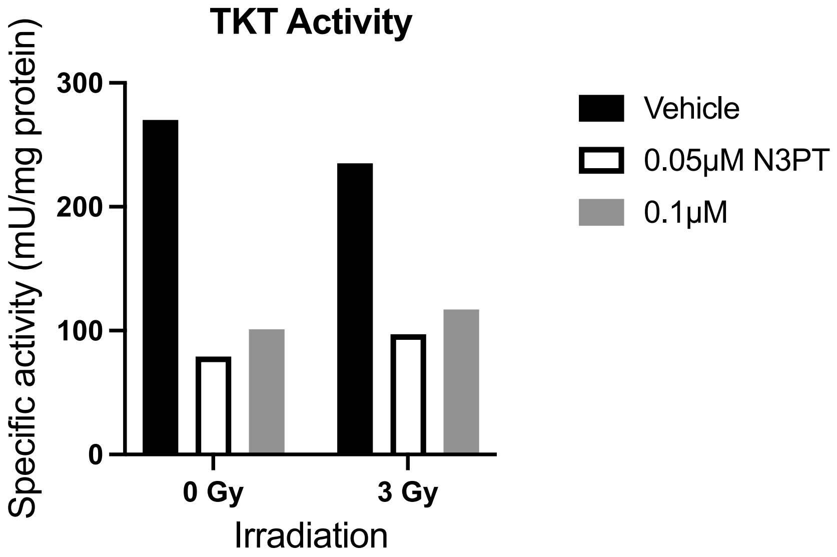

Supplemental Figure 2. N3PT reduces TKTL activity in HLFs. HLFs were seeded in 12 well dishes and irradiated as previously described, cultured for 5 days, then lysed according to the TKT assay kit directions. N3PT in 0.1% DMSO, or DMSO control, was added to the assay 10 minutes prior to adding the rest of the assay mix, and TKT activity was determined. Results shown are the average of two technical replicates of single cell cultures.

Supplemental Figure S4

A.

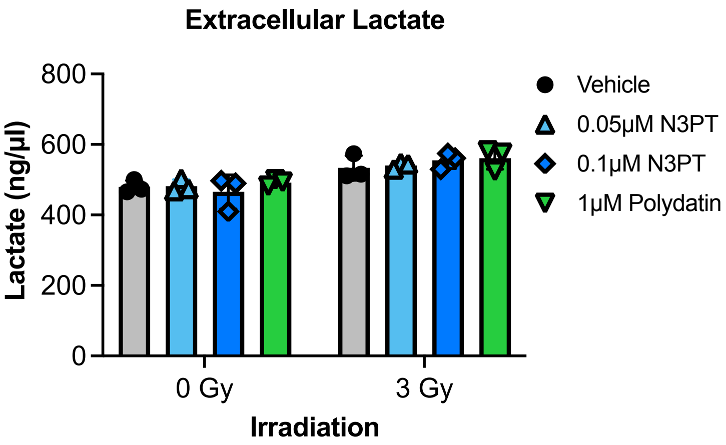

B.

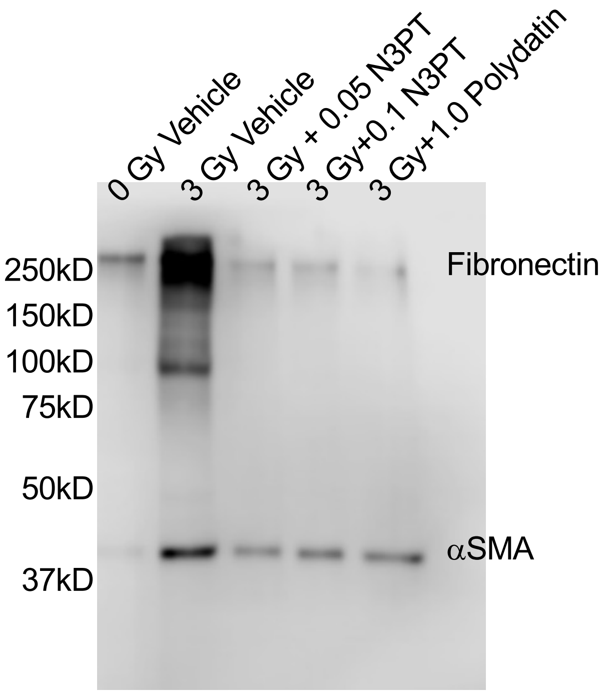

Supplemental Figure 4. N3PT and polydatin do not affect extracellular lactate. HLFs were seeded in 12 well dishes and irradiated as previously described. N3PT (0.05µM or 0.1µM in 0.1% DMSO), polydatin (1µM in DMSO) or DMSO control was added and the HLFs were cultured for 5 days**. A.** Conditioned medium was collected and lactate was measured with a commercial assay kit. **B.** The cells were lysed, and expression of fibronectin and αSMA was analyzed by western blot. One representative lane per condition is shown for 3 replicates per condition.
